# Neuronal Induction of Bone‐Fat Imbalance through Osteocyte Neuropeptide Y

**DOI:** 10.1002/advs.202100808

**Published:** 2021-10-31

**Authors:** Yan Zhang, Chun‐Yuan Chen, Yi‐Wei Liu, Shan‐Shan Rao, Yi‐Juan Tan, Yu‐Xuan Qian, Kun Xia, Jie Huang, Xi‐Xi Liu, Chun‐Gu Hong, Hao Yin, Jia Cao, Shi‐Kai Feng, Ze‐Hui He, You‐You Li, Zhong‐Wei Luo, Ben Wu, Zi‐Qi Yan, Tuan‐Hui Chen, Meng‐Lu Chen, Yi‐Yi Wang, Zhen‐Xing Wang, Zheng‐Zhao Liu, Ming‐Jie Luo, Xiong‐Ke Hu, Ling Jin, Teng‐Fei Wan, Tao Yue, Si‐Yuan Tang, Hui Xie

**Affiliations:** ^1^ Department of Orthopedics Xiangya Hospital Central South University Changsha Hunan 410008 China; ^2^ Movement System Injury and Repair Research Center Xiangya Hospital Central South University Changsha Hunan 410008 China; ^3^ Department of Pediatrics Union Hospital Tongji Medical College Huazhong University of Science and Technology Wuhan 430022 China; ^4^ Department of Sports Medicine Xiangya Hospital Central South University Changsha Hunan 410008 China; ^5^ Xiangya School of Nursing Central South University Changsha Hunan 410013 China; ^6^ Department of Neurology Xiangya Hospital Central South University Changsha Hunan 410013 China; ^7^ National Clinical Research Center for Geriatric Disorders Xiangya Hospital Central South University Changsha Hunan 410008 China; ^8^ Hunan Key Laboratory of Organ Injury Aging and Regenerative Medicine Xiangya Hospital Central South University, Changsha Changsha Hunan 410008 China; ^9^ Hunan Key Laboratory of Bone Joint Degeneration and Injury Xiangya Hospital Central South University, Changsha Changsha Hunan 410008 China

**Keywords:** adipogenesis, autonomic nervous system, bone marrow mesenchymal stem/stromal cells, neuropeptide Y, osteocyte, osteogenesis

## Abstract

A differentiation switch of bone marrow mesenchymal stem/stromal cells (BMSCs) from osteoblasts to adipocytes contributes to age‐ and menopause‐associated bone loss and marrow adiposity. Here it is found that osteocytes, the most abundant bone cells, promote adipogenesis and inhibit osteogenesis of BMSCs by secreting neuropeptide Y (NPY), whose expression increases with aging and osteoporosis. Deletion of NPY in osteocytes generates a high bone mass phenotype, and attenuates aging‐ and ovariectomy (OVX)‐induced bone‐fat imbalance in mice. Osteocyte NPY production is under the control of autonomic nervous system (ANS) and osteocyte NPY deletion blocks the ANS‐induced regulation of BMSC fate and bone‐fat balance. *γ*‐Oryzanol, a clinically used ANS regulator, significantly increases bone formation and reverses aging‐ and OVX‐induced osteocyte NPY overproduction and marrow adiposity in control mice, but not in mice lacking osteocyte NPY. The study suggests a new mode of neuronal control of bone metabolism through the ANS‐induced regulation of osteocyte NPY.

## Graphical abstract

Normally, norepinephrine (NE) and acetylcholine (ACh) production is maintained at a balanced level in bone, so that osteocytes cannot generate excessive neuropeptide Y (NPY) to favor bone marrow mesenchymal stem/stromal cell adipogenesis rather than osteogenesis. With aging/estrogen deficiency, sympathetic overactivity, and decreased parasympathetic activity cause NE overproduction and ACh reduction, resulting in excess osteocyte NPY generation and subsequent bone‐fat imbalance.

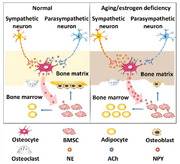

## 1 Introduction

Bone remodeling occurs throughout life and skeleton integrity is maintained through the coupled action of bone‐forming osteoblasts and bone‐resorbing osteoclasts.^[^ ^1^ ^]^ An imbalance of this coupling leads to multiple bone diseases, of which the most common is osteoporosis, a disabling disorder frequently affecting the elderly people especially postmenopausal women.^[^ ^2^ ^]^ Bone marrow adipose tissue accumulates with skeletal aging and menopause at the expense of bone formation, which occurs partly due to a shift of the differentiation of bone marrow mesenchymal stem/stromal cells (BMSCs) towards adipocytes rather than osteoblasts and finally causes osteoporosis.^[^ ^3^, ^4^ ^]^ Thus, deciphering the mechanism that controls BMSC fate switching is beneficial for developing strategies against skeletal aging and osteoporosis.

Osteocytes are the most abundant (>90%) bone cells embedded in the mineralized matrix, where they form a neuron‐like connective network to communicate with each other and with osteoblasts and osteoclasts on the bone surface (BS).^[^ ^5^, ^6^ ^]^ Osteocytes act as mechanosensors that transduce mechanical stimuli to biochemical signals to affect the surrounding cells.^[^ ^5^, ^7^ ^]^ They secrete sclerostin that inhibits osteoblast activity and bone formation,^[^ ^8^ ^]^ but also serve as a source of Wnt1 ligand to stimulate osteogenesis.^[^ ^9^ ^]^ Osteocytes are also able to secrete anti‐ or pro‐osteoclastogenic molecules to regulate osteoclastic bone resorption.^[^ ^10^, ^11^ ^]^ The double‐faced role of osteocytes may be attributed to that their regulation occurs in a temporal‐ and spatial‐dependent manner. Currently, evidence is lacking on whether osteocytes, the major player in the bone, have the ability to contact with BMSCs to modulate their differentiation fate.

Neuropeptide Y (NPY) is a highly conserved 36‐amino‐acid peptide usually abundant in the central nervous system (CNS).^[^ ^12^ ^]^ The signals of NPY are mediated by five receptors (Y1R, Y2R, Y4R, Y5R, and Y6R), among which Y1R and Y2R have roles in regulating bone mass. In the CNS, hypothalamic NPY likely acts through Y2R to inhibit bone formation.^[^ ^13^ ^]^ In the periphery, NPY can be released from the sympathetic nerves^[^ ^14^ ^]^ and adrenal medulla.^[^ ^15^ ^]^ More importantly, NPY is demonstrated to be abundantly expressed in the bone tissue predominantly by osteocytes and at a much lower level by osteoblasts.^[^ ^16^ ^]^ A direct inhibitory role of NPY in BMSC proliferation and osteoblast differentiation through Y1R has been reported,^[^ ^16^, ^17^ ^]^ suggesting a local role of NPY in the regulation of bone metabolism. These findings motivated us to investigate whether osteocytes are capable of functioning through NPY to affect BMSC fate and bone‐fat balance.

Another important question is which signaling controls osteocyte NPY production. The autonomic nervous system (ANS) with its two antagonistic branches, the sympathetic nervous system (SNS) and the parasympathetic nervous system (PSNS), communicates with bone to regulate its homeostasis.^[^ ^18^ ^]^ SNS overactivity is a hallmark of aging^[^ ^19^ ^]^ and leads to bone formation inhibition and bone loss through *β*‐adrenergic receptors (*β*ARs).^[^ ^18^, ^20^, ^21^ ^]^ In contrast to SNS, PSNS positively regulates bone mass and a decrease of PSNS activity is associated with osteoporosis.^[^ ^18^, ^22^, ^23^ ^]^ It is unclear whether SNS and PSNS have regulatory roles in BMSC fate decisions and bone‐fat balance. Skeleton sympathetic and parasympathetic innervation has been indicated by the presence of immune‐positive neuronal fibers for the SNS marker tyrosine hydroxylase (TH) and PSNS marker vesicular acetylcholine transporter (VaChT) in periosteum, mineralized bone, or/and bone marrow.^[^ ^18^, ^24^ ^]^ In the sympathetic nerves, NPY is co‐stored with norepinephrine (NE) and released upon SNS activation.^[^ ^14^ ^]^ These reports, along with the evidence of the presence of NE transporter and *β*2AR in osteocytes^[^ ^25^, ^26^ ^]^ prompted us to explore whether correlations exist among ANS, osteocytes, and NPY.

Herein, we report that during aging and osteoporosis, osteocytes secrete excess NPY to promote adipogenic differentiation of BMSCs at the expense of osteogenesis through Y1R. We identify the downstream effectors that are modulated by osteocyte NPY and mediate the osteocyte NPY‐induced regulation of BMSC fate. Furthermore, we demonstrate that the mice lacking osteocyte NPY exhibit a high bone mass phenotype and significant reduction of aging‐ and ovariectomy (OVX)‐associated bone loss and marrow fat accumulation. Osteocyte NPY production is regulated by ANS through *β*2AR and muscarinic acetylcholine (ACh) receptor M3R, and is required for the ANS‐induced regulation of BMSC differentiation and bone‐fat balance. *γ*‐Oryzanol, a rice bran‐derived component that has been clinically used against autonomic dysfunction syndromes,^[^ ^27^ ^]^ effectively inhibits bone NPY production and protects against aging‐ and OVX‐induced bone loss and marrow adiposity in control mice, but these effects are not obvious in osteocyte NPY‐lacking mice. Our study provides the first evidence that osteocytes function as a mediator of neuronal control of BMSC fate decisions and bone‐fat balance through the secretion of NPY.

## 2 Results

### 2.1 Osteocytes Secrete Excess NPY during Skeletal Aging and Osteoporosis to Shift BMSC Fate from Osteogenesis towards Adipogenesis via Y1R

**Figure 1:**
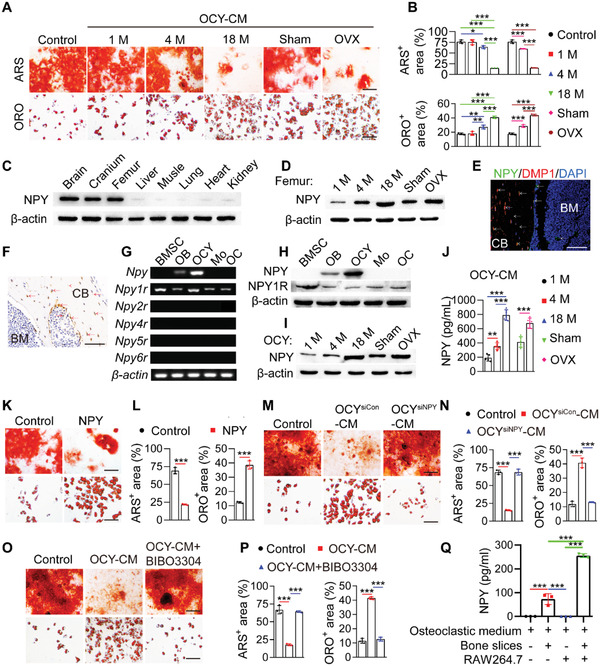
Osteocytes secrete excess NPY during skeletal aging and osteoporosis to shift BMSC fate from osteogenesis towards adipogenesis via Y1R. A) ARS and ORO staining images of BMSCs treated with the culture media (OCY‐CM) from different donor mice‐derived osteocytes or un‐cultured medium (Control) under osteogenic or adipogenic induction. OVX: ovariectomy. Scale bar: 50 µm. B) Quantitation of the percentages of ARS^+^ and ORO^+^ areas. *n* = 3 per group. Western blotting for NPY protein C) in different tissues from 4‐month‐old male wild‐type mice, or D) in the bone marrow‐depleted femurs from normal male mice at different ages or female mice subjected to OVX or sham operation. NPY expression in the bone from 4‐month‐old male wild‐type mice was tested by E) immunofluorescence double staining for DMP1/NPY and F) immunohistochemical staining for NPY. Arrows indicate the representative NPY^+^/DMP1^+^ or NPY^+^ signals. BM: bone marrow; CB: cortical bone. Scale bar: 50 µm. G) Semiquantitative PCR analysis of mRNA expression of NPY and its five receptors, and H) western blotting for NPY protein in different bone and bone marrow cell types from 8‐month‐old male wild‐type mice. OB: osteoblast; OCY: osteocyte; Mo: monocyte/macrophage; OC: osteoclast. NPY protein in the osteocyte lysates and OCY‐CM was measured by I) western blotting and J) ELISA, respectively. *n* = 5 per group. K) ARS and ORO staining images and L) quantification of the positively stained areas in BMSCs treated with vehicle or NPY protein under osteogenic or adipogenic induction. Scale bar: 50 µm. *n* = 3 per group. M,N) Knockdown of NPY in osteocytes using siRNAs blocked the capacities of their culture media (OCY^siNPY^‐CM) to inhibit osteogenesis and promote adipogenesis of BMSCs. Scale bar: 50 µm. *n* = 3 per group. O, P) Blockade of Y1R on the differentiating BMSCs by the Y1R antagonist BIBO3304 abolished the anti‐osteogenic and pro‐adipogenic effects of OCY‐CM. Scale bar: 50 µm. *n* = 3 per group. Q) ELISA for NPY protein. *n* = 3 per group. Data are presented as mean ± SD. For panels (L) and (P): unpaired, two‐tailed student's *t*‐test. For other dot plots: one‐way ANOVA with Bonferroni post hoc test. *P < 0.05, **P < 0.01, and ***P < 0.001.

First, we examined the effects of supernatants of osteocyte cultures on osteogenic and adipogenic differentiation of mouse primary BMSCs in vitro. Osteocytes were identified by optical microscope observation, immunofluorescence staining for dentin matrix protein 1 (DMP1), and alkaline phosphatase (ALP) staining. The results showed that osteocytes exhibited a stellate morphology with long dendritic processes, expressed DMP1 protein, but did not produce ALP protein (Figure S1A, Supporting Information). The identity of BMSCs was determined by the murine MSC surface marker expression profiles (Sca‐1^+^, CD44^+^, CD90^+^, CD45^−^, and CD34^−^) and the ability to differentiate toward osteoblasts and adipocytes, as indicated by surface marker analysis using flow cytometry (Figure S1B, Supporting Information) and multi‐potent differentiation potential assessment using Alizarin Red S (ARS) and Oil Red O (ORO) staining (Figure S1C, Supporting Information). ARS and ORO staining, respectively, revealed that incubation with the culture media from 4‐month‐old normal mice‐ or 3.5‐month‐old sham‐operated mice‐derived osteocytes (OCY‐CM) induced a statistically significant suppression of osteogenesis and upregulation of adipogenesis of BMSCs (**Figure** **1**A,B). With the age increase of the donor mice or once the mice underwent OVX surgery, the anti‐osteogenic and pro‐adipogenic activities of their OCY‐CM were further augmented (Figure 1A,B), suggesting that osteocytes may be an important contributor to bone‐fat imbalance during skeletal aging and estrogen deficiency‐induced osteoporosis.

Western blotting confirmed the much higher level of NPY protein in the brain, calvaria, and femur compared with other organs in 4‐month‐old wild‐type mice (Figure 1C). More importantly, we found that the protein level of NPY in the bone tissue was dramatically increased with aging and with the induction of osteoporosis by OVX surgery in young mice (Figure 1D), implying that NPY may be involved in regulating bone homeostasis in skeletal aging and osteoporosis. In the brain, NPY production was decreased with aging and not obviously changed after OVX (Figure S2, Supporting Information).

We then performed immunofluorescence double staining for DMP1/NPY and immunohistochemical staining for NPY to detect NPY expression in the mouse bone. Double staining for DMP1/NPY showed that NPY protein was abundantly detected in the DMP1‐positive osteocytes within the bone matrix. NPY protein was also presented in the site of bone matrix, many cells at the BS, and a few cells in the bone marrow (Figure 1E). This was consistent with the result of immunohistochemical staining for NPY in Figure 1F, which confirmed the presence of NPY protein in osteocytes within the bone matrix, osteoblasts at the BS, the area of bone matrix, and some cells in the bone marrow. We isolated mouse osteocytes, osteoblasts, BMSCs, and monocytes/macrophages, and then assessed NPY expression in these cells as well as in osteoclasts differentiated from monocytes/macrophages. The identification results of osteocytes and BMSCs were shown in Figure S1A–S1C, Supporting Information. Osteoblasts were characterized by the expression of OCN and the ability to produce ALP (Figure S3A, Supporting Information). The identities of monocytes/macrophages and osteoclasts were determined by flow cytometry and tartrate‐resistant acid phosphatase (TRAP) staining, respectively, which showed that monocytes/macrophages co‐expressed F4/80 and CD11b (Figure S3B, Supporting Information), and osteoclasts were positive for TRAP (Figure S3C, Supporting Information). Semiquantitative polymerase chain reaction (PCR) and western blotting showed the abundant expression of NPY in osteocytes at the mRNA and protein levels, respectively (Figure 1G,H). A much lower level of NPY expression was observed in osteoblasts, but not in BMSCs, monocytes/macrophages, and osteoclasts (Figure 1G,H). Among the five NPY receptors, only *Npy1r* mRNA and NPY1R protein were abundantly detected in bone or bone marrow cells especially BMSCs (Figure 1G,H), suggesting that BMSCs are the primary target of osteocyte NPY. The expression of Y1R protein in osteocytes, osteoblasts, and cells in the bone marrow was confirmed by immunohistochemical staining (Figure S4A, Supporting Information). The abundant expression of Y1R in BMSCs was further verified by immunostaining for this receptor (Figure S4B, Supporting Information). An age‐dependent and OVX‐induced increase of NPY production was observed in osteocytes (Figure 1I) and OCY‐CM (Figure 1J). These results suggest that osteocytes may act through NPY/Y1R signaling to affect BMSC fate decisions.

The direct role of recombinant NPY protein in inhibiting osteogenesis and promoting adipogenesis of BMSCs was shown by ARS and ORO staining (Figures 1K,L) and quantitative real‐time PCR (qRT‐PCR) for the genes related to osteogenesis (*Runx2*, *Bglap*, *Alpl*, and *Postn*) or adipogenesis (*Pparγ*, *Fabp4*, and *Cebpα*) (Figure S5A,S5B, Supporting Information). To determine the essential role of NPY in the osteocytes‐induced regulation of BMSC fate, we down‐regulated *Npy* expression in osteocytes from 8‐month‐old mice using specific siRNAs, whose inhibitory efficiency was confirmed by qRT‐PCR (Figure S5C, Supporting Information), or interfered with Y1R on the differentiating BMSCs using the Y1R antagonist BIBO3304. As indicated by ARS and ORO staining, both of these treatments effectively abolished the anti‐osteogenic and pro‐adipogenic effects of OCY‐CM on BMSCs (Figure 1M–P). Collectively, these data indicate that NPY mediates the osteocytes‐induced regulation of BMSC differentiation fate through Y1R.

To explore whether NPY can be released from bone during osteoclastic bone resorption, an in vitro assay was conducted to mimic this process. The osteoclast progenitor RAW264.7 cells were cultured in osteoclastic induction medium with or without bone slices from 4‐month‐old wild‐type mice. Large numbers of osteoclasts were formed after osteoclastic induction for 8 days and the cells were cultured in a fresh serum‐free osteoclastic medium for another 48 h, in order to test the concentration of NPY protein by enzyme‐linked immunosorbent assay (ELISA). The result showed no presence of NPY protein in both conditioned medium (CM) from osteoclasts and osteoclastic induction medium without cells and bone slices (Figure 1Q). However, NPY protein could be detected in CM from osteoclastic induction medium + bone slices group, and the concentration of NPY was significantly increased in CM from RAW264.7 cells + osteoclastic induction medium + bone slices group (Figure 1Q), indicating that the cultured osteoclasts‐induced bone resorption results in the release of a large amount of NPY protein into the culture medium. The result suggests that osteocyte NPY can be released from bone to the bone marrow during osteoclastic bone resorption.

### 2.2 Deletion of NPY in Osteocytes Attenuates Aging‐ and OVX‐Associated Bone Loss and Marrow Adiposity

**Figure 2:**
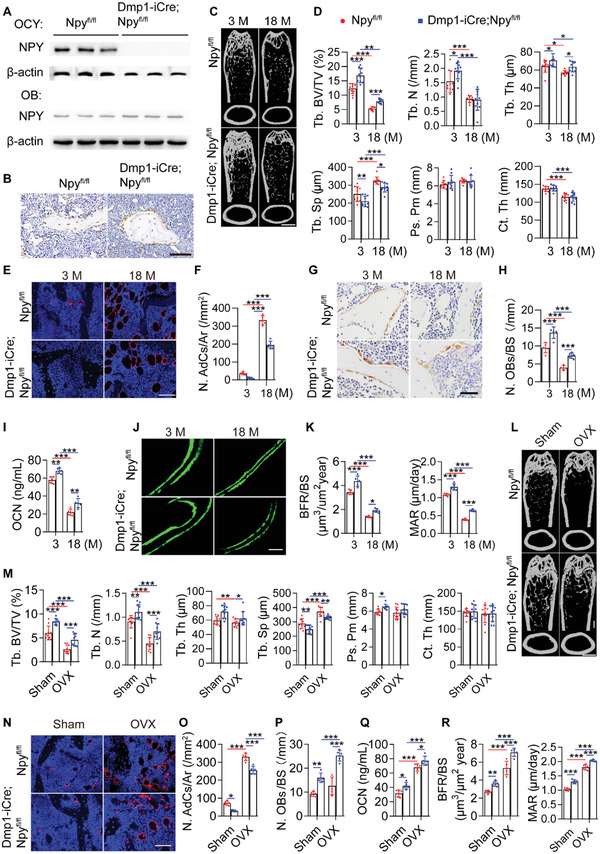
Deletion of NPY in osteocytes attenuates aging‐ and OVX‐induced bone loss and marrow adiposity. A) Western blotting for NPY protein in osteocytes and osteoblasts from 4‐month‐old male *Npy^fl/fl^* mice and *Dmp1‐iCre*; *Npy^fl/fl^* mice. B) Immunohistochemical staining for NPY in the bone tissues. Scale bar: 50 µm. C) µCT reconstruction images and D) quantification of bone microarchitecture parameters in femurs from aged‐matched male *Npy^fl/fl^* mice and *Dmp1‐iCre*; *Npy^fl/fl^* mice. Tb. BV/TV: trabecular bone volume fraction; Tb. N: trabecular number; Tb. Th: trabecular thickness; Tb. Sp: trabecular separation; Ps. Pm: periosteal perimeter; Ct. Th: cortical thickness. Scale bar: 1 mm. *n* = 10 per group. E) Immunofluorescence staining images for perilipin and F) quantification of adipocyte number in distal femurs. Scale bar: 50 µm. *n* = 5 per group. G) OCN immunohistochemical staining images and H) the number of OCN‐stained osteoblasts (N. OBs) on trabecular BS in distal femurs. Scale bar: 25 µm. *n* = 5 per group. I) ELISA for serum OCN. *n* = 5 per group. J) Calcein double labeling of trabecular bones and K) quantification of BFR/BS and MAR. Scale bar: 25 µm. *n* = 5 per group. L) µCT reconstruction images and M) quantification of bone microarchitecture parameters in femurs from sham‐ or OVX‐operated female *Npy^fl/fl^* mice and *Dmp1‐iCre*; *Npy^fl/fl^* mice. Scale bar: 1 mm. *n* = 10 per group. N) Immunostaining images for perilipin in distal femurs and O) quantification of adipocyte number. Scale bar: 50 µm. *n* = 5 per group. P) Quantification of OCN^+^ osteoblast number in distal femurs. *n* = 5 per group. Q) ELISA for serum OCN. *n* = 5 per group. R) Quantification of BFR/BS and MAR. *n* = 3 per group. Data are presented as mean ± SD. Two‐way ANOVA combined with Bonferroni post hoc test. *P < 0.05, **P < 0.01, and ***P < 0.001.

We next generated *Npy^fl/fl^* mice harboring a *Npy* allele in which exon 2 was flanked by two *loxP* sites and crossed *Npy^fl/fl^* mice with transgenic mice expressing improved Cre recombinase under the control of the *Dmp1* gene promoter (*Dmp1‐iCre*) to generate osteocyte NPY‐deleted (*Dmp1‐iCre*; *Npy^fl/fl^*) mice (Figure S6A,S6B, Supporting Information). NPY protein expression was much lower in the bone marrow‐depleted femur homogenates, but not in the whole bone marrow cells, the whole brain lysate, and the NPY‐abundant hypothalamus, of 4‐month‐old *Dmp1‐iCre*; *Npy^fl/fl^* mice than that of *Npy^fl/fl^* littermates (Figure S6C, Supporting Information). The comparable level of hypothalamic NPY protein between *Npy^fl/fl^* mice and *Dmp1‐iCre*; *Npy^fl/fl^* mice was further confirmed by immunofluorescence staining for NPY in the brain tissues (Figure S6D,S6E, Supporting Information). Few DMP1/NPY‐double ‐positive cells in the brain (Figures S7, Supporting Information) could account for why there was no significant difference of NPY expression in the whole brain and hypothalamus between *Dmp1‐iCre*; *Npy^fl/fl^* mice and *Npy^fl/fl^* mice. NPY deletion in osteocytes rather than in osteoblasts of *Dmp1‐iCre*; *Npy^fl/fl^* mice, was confirmed by western blotting for the protein lysates of the isolated osteocytes and osteoblasts (**Figure** **2**A). Immunohistochemical staining was further conducted to assess NPY expression in the bone tissues. Consistently, the result showed that NPY protein was absent in osteocytes, but still observed in many osteoblasts of *Dmp1‐iCre*; *Npy^fl/fl^* mice (Figure 2B).

Microcomputed tomography (µCT) analysis of the femurs revealed that 3‐month‐old *Dmp1‐iCre*; *Npy^fl/fl^* mice displayed a high trabecular bone mass phenotype, as indicated by much higher levels of trabecular bone volume fraction (Tb. BV/TV), trabecular number (Tb. N), and trabecular thickness (Tb. Th), as well as lower level of trabecular separation (Tb. Sp) than those of *Npy^fl/fl^* mice, *Dmp1‐iCre* mice or/and wild‐type mice of the same age (Figure 2C,D; Figure S8A,8B, Supporting Information). For cortical bone mass, deletion of osteocyte NPY induced a trend increase of periosteal perimeter (Ps. Pm), and no obvious changes were observed in the level of cortical thickness (Ct. Th) among these groups (Figure 2C,D; Figure S8A,S8B, Supporting Information). Age‐dependent loss of bone mass, adipocyte accumulation in marrow, reduction of osteogenic activity, and decrease of new bone formation capacity were all significantly attenuated in *Dmp1‐iCre*; *Npy^fl/fl^* mice compared with the age‐matched *Npy^fl/fl^* mice, as indicated by µCT analysis (Figure 2C,D), immunofluorescence staining for perilipin (Figure 2E,F), quantification of the number of osteocalcin (OCN)‐stained osteoblasts (Figure 2G,H) and the serum level of OCN (Figure 2I), as well as bone formation rate per BS (BFR/BS) and mineral apposition rate (MAR) values using double calcein labeling (Figure 2J,K), respectively. We also tested whether osteocyte‐lacking NPY affects bone loss and marrow adiposity in OVX mice. Although deletion of NPY in osteocytes did not abolish the OVX‐induced loss of bone mass and marrow fat accumulation, the ovariectomized *Dmp1‐iCre*; *Npy^fl/fl^* mice showed significantly higher trabecular bone mass (Figure 2L,M), a trend of increase of cortical bone mass (Figure 2L,M), fewer bone marrow adipocytes (Figure 2N,O), and higher levels of parameters revealing osteogenic activity (Figure 2P,Q) and new bone formation/mineralization (Figure 2R), as compared with the ovariectomized *Npy^fl/fl^* mice. These results indicate that deletion of NPY in osteocytes alleviates aging‐ and OVX‐induced bone loss and marrow adiposity.

The number of osteoclasts just showed a trend of increase in the aged (18‐month‐old) mice compared with the 3‐month‐old adult mice, but osteoclast activity was markedly augmented with aging, as revealed by TRAP staining (Figure S8C,S8D, Supporting Information) and ELISA for the serum bone resorption marker C‐terminal telopeptides of type I collagen (CTX‐I; Figure S8E, Supporting Information). However, both osteoclast formation and activity were profoundly enhanced after OVX (Figure S8F–S8H, Supporting Information). Osteocyte‐specific deletion of NPY did not notably affect osteoclast formation and activity in the adult, aged, and OVX mice (Figure S8C–S8H, Supporting Information). This was consistent with the in vitro data showing no obvious effect of NPY on osteoclast formation of osteoclast progenitor RAW264.7 cells (Figure S8I,S8J, Supporting Information).

NPY represents a potent orexigenic factor in the hypothalamus.^[^ ^28^ ^]^ In the periphery, NPY has been reported to stimulate preadipocyte differentiation and abdominal fat growth.^[^ ^29^ ^]^ However, there were no significant differences in the values of body weight (Figure S8K, Supporting Information), lean mass (Figure S8L, Supporting Information), fat mass (Figure S8M, Supporting Information), and daily consumptions of food and water (Figure S8N,S8O, Supporting Information) between age‐matched female *Dmp1‐iCre*; *Npy^fl/fl^* mice and *Npy^fl/fl^* mice, consistent with the result showing a similar protein level of NPY in the hypothalamus of these mice. We also measured the values of serum total protein, albumin, urea nitrogen, creatinine, blood glucose, total triglyceride, and total cholesterol in 3‐month‐old female *Dmp1‐iCre*; *Npy^fl/fl^* mice and *Npy^fl/fl^* mice, in order to evaluate the metabolic status of these mice. Consistently, no remarkable changes were detected in these parameters between *Dmp1‐iCre*; *Npy^fl/fl^* mice and *Npy^fl/fl^* mice (Figure S8P–S8V, Supporting Information). These findings suggest that hypothalamic NPY is not notably affected in *Dmp1‐iCre*; *Npy^fl/fl^* mice and osteocyte NPY appears to act locally within bone tissue as a promotor of bone‐fat imbalance.

Since osteoblasts also express NPY in the bone tissues, we then evaluated whether deletion of NPY in osteoblasts is capable of altering bone mass in mice. Collagen type I alpha 1 (COL1a1) is abundantly expressed by osteoblasts at embryonal and post‐natal stages.^[^ ^30^ ^]^ *Col1a1‐Cre* has been frequently used to delete genes in osteoblasts.^[^ ^31^ ^]^ Since osteocytes are differentiated from osteoblasts, these genes can be also deleted in osteocytes newly generated from osteoblasts after *Col1a1‐Cre* expression. We crossed *Npy^fl/fl^* mice with *Col1a1‐CreERT2* mice that express a tamoxifen‐inducible Cre recombinase driven by the *Col1a1* promoter, in order to generate *Col1a1‐Cre/ERT2*; *Npy^fl/fl^* mice (Figure S9A, Supporting Information). After tamoxifen induction, NPY could be deleted in osteoblasts of *Col1a1‐Cre/ERT2*; *Npy^fl/fl^* mice. NPY would be also deleted in a minority of osteocytes that were newly transformed from the NPY‐deleted osteoblasts, but present in osteocytes that had been embedded in the bone matrix before the use of tamoxifen. µCT analysis of the femurs showed that *Col1a1‐Cre/ERT2*; *Npy^fl/fl^* mice treated with tamoxifen at adult stage just exhibited a significantly lower value of Tb. Sp compared with the tamoxifen‐treated *Npy^fl/fl^* mice of the same age (Figure S9B,S9C, Supporting Information). Tb. BV/TV, Tb. N, Tb. Th, and Ct. Th were increased in the tamoxifen‐treated *Col1a1‐Cre/ERT2*; *Npy^fl/fl^* mice compared to *Npy^fl/fl^* mice receiving tamoxifen, but only by trend (Figure S9B,S9C, Supporting Information). These data suggest that depletion of NPY in osteoblasts and a few osteocytes is not sufficient to induce significant effects on bone mass.

### 2.3 The Impact of Osteocyte NPY Deletion on the Ability of OCY‐CM to Regulate BMSC Fate and the Differentiation Potential of BMSCs

**Figure 3:**
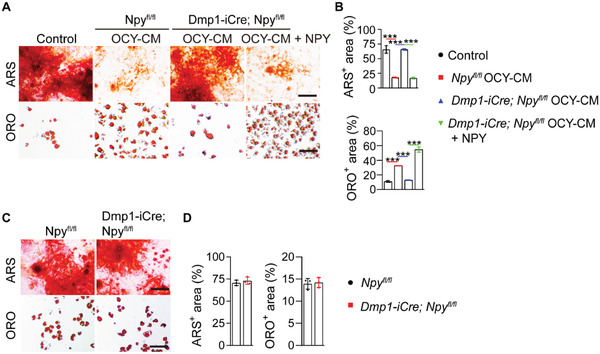
The impact of osteocyte NPY deletion on the ability of OCY‐CM to regulate BMSC fate and on the differentiation potential of BMSCs. A) ARS and ORO staining images and B) quantification of the positively stained areas showing the effects of OCY‐CM from *Npy^fl/fl^* mice and *Dmp1‐iCre*; *Npy^fl/fl^* mice on BMSC differentiation towards osteogenesis and adipogenesis, respectively. Scale bar: 50 µm. *n* = 3 per group. C) ARS and ORO staining images and D) quantification of the positively stained areas showing the in vitro osteogenic and adipogenic differentiation potential of BMSCs from *Npy^fl/fl^* mice and *Dmp1‐iCre*; *Npy^fl/fl^* mice. Scale bar: 50 µm. *n* = 3 per group. Data are presented as mean ± SD. For panel (B): unpaired, two‐tailed student's *t*‐test (differences between *Dmp1‐iCre*; *Npy^fl/fl^* OCY‐CM and *Dmp1‐iCre*; *Npy^fl/fl^* OCY‐CM + NPY groups) or one‐way ANOVA combined with Bonferroni post hoc test (differences among other groups except *Dmp1‐iCre*; *Npy^fl/fl^* OCY‐CM + NPY group). For panel (D): unpaired, two‐ailed student's *t*‐test. ***P < 0.001.

Unlike OCY‐CM from *Npy^fl/fl^* mice, the *Dmp1‐iCre*; *Npy^fl/fl^* mice‐derived OCY‐CM lost the ability to inhibit osteogenesis and promote adipogenesis of BMSCs, but the deficits were rescued with the addition of NPY protein (**Figure** **3**A,B), which further confirmed the necessary role of NPY in the osteocytes‐induced regulation of BMSC differentiation. No obvious differences were found in the in vitro osteogenic and adipogenic differentiation abilities of BMSCs from *Dmp1‐iCre*; *Npy^fl/fl^* mice and *Npy^fl/fl^* mice (Figure 3C,D), suggesting that the osteocytes‐induced alterations in the bone microenvironment, but not the changes of the differential potential of BMSCs themselves, are critical for BMSC lineage switching and bone‐fat imbalance in vivo.

### 2.4 Osteocyte NPY‐Induced BMSC Fate Switching is Mediated by the Inhibition of TEAD1 and JUNB Through cAMP/PKA/CREB Signaling

**Figure 4:**
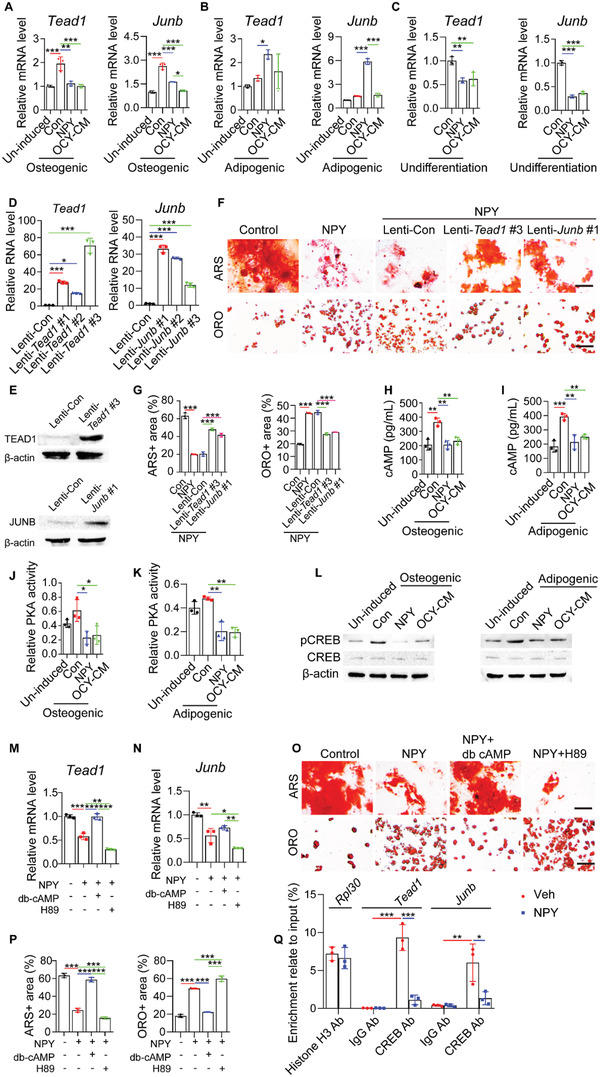
Osteocyte NPY‐induced BMSC fate switching is mediated by the inhibition of TEAD1 and JUNB through cAMP/PKA/CREB signaling. qRT‐PCR analysis of *Tead1* and *Junb* expression in BMSCs receiving different treatments under A) osteogenic or B) adipogenic induction for 24 h, or C) in undifferentiated BMSCs with different treatments for 24 h. *n* = 3 per group. D) qRT‐PCR and E) western blotting confirmed the overexpression efficiency of recombinant *Tead1* and *Junb* lentiviruses in BMSCs. *n* = 3 per group. F) ARS and ORO staining images and G) quantification of the positively stained areas showing the reduced activity of NPY to inhibit osteogenesis and promote adipogenesis of BMSCs overexpressing *Tead1* or *Junb*. Scale bar: 50 µm. *n* = 3 per group. ELISA for cAMP in BMSCs receiving different treatments under H) osteogenic or I) adipogenic induction for 24 h. *n* = 3 per group. PKA activity assay for cell homogenates of BMSCs receiving different treatments under J) osteogenic or K) adipogenic induction for 24 h. *n* = 3 per group. L) Western blotting for CREB and phosphorylated CREB (p‐CREB) in BMSCs receiving different treatments under osteogenic or adipogenic induction for 24 h. qRT‐PCR analysis of M) *Tead1* and N) *Junb* expression in BMSCs receiving different treatments under osteogenic induction for 24 h. *n* = 3 per group. O) ARS and ORO staining images and P) quantification of the positively stained areas in BMSCs receiving different treatments under osteogenic or adipogenic induction. Scale bar: 50 µm. *n* = 3 per group. Q) ChIP‐qRT‐PCR analysis shows enrichment of the CREB antibody (CREB Ab)‐immunoprecipitated *Tead1* or *Junb* promoter region relative to the input DNA. Normal rabbit anti‐IgG served as a negative control. Histone H3 antibody (Histone H3 Ab) pulldown for the enrichment of *Rpl30* gene served as a positive control. *n* = 3 per group. Data are presented as mean ± SD. For panels (A), (B), (G), (H–K), (M), (N), and (P): unpaired, two‐tailed student's *t*‐test (differences between un‐induced and control groups in (A), (B), and (H–K), or between control and NPY groups in (G), (M), (N), and (P)) or one‐way ANOVA combined with Bonferroni post hoc test (differences among other groups except un‐induced group in (A), (B), and (H–K), or except control group in (M), (N), and (P)). For panel (C) and (D): one‐way ANOVA combined with Bonferroni post hoc test. For panel (Q): unpaired, two‐tailed student's *t*‐test (differences between control and NPY groups for *Rpl30*) or two‐way ANOVA combined with Bonferroni post hoc test (differences among other groups for *Tead1* or *Junb*). *P < 0.05, **P < 0.01, ***P < 0.001.

We further explored the downstream factors associated with osteocyte NPY‐induced BMSC differentiation. More recently, Rauch et al. identified that osteoblast lineage determination of BMSCs is driven by the concerted action of a subset of transcription factors (*Snai2*, *Mef2a*, *Tead1*, *Tead4*, *Smad3*, *Hif1a*, *Elk4*, *Pitx1*, and *Junb*) that are expressed in undifferentiated BMSCs and act as stimulators of osteogenesis or/and molecular brakes on adipogenesis.^[^ ^32^ ^]^ Knockdown of a single member of this network is sufficient to repress osteogenesis or/and accelerate adipogenesis of BMSCs.^[^ ^32^ ^]^ Thus, we focused on these transcription factors to explore the molecular mechanism by which osteocyte NPY affects BMSC fate decisions. The involvement of TAZ, a transcriptional modulator that acts as a co‐activator of RUNX2 to promote BMSC osteogenesis while as a co‐repressor of PPAR*γ* to suppress BMSC adipogenesis,^[^ ^3^, ^33^ ^]^ was also investigated. qRT‐PCR showed that osteogenic induction for 24 h induced significant increases in the expression of most of these pro‐osteogenic/anti‐adipogenic transcription factors in BMSCs, whereas only the expression of *Snai2*, *Mef2a*, *Tead1*, and *Junb* was markedly inhibited by both NPY and OCY‐CM (**Figure** **4**A; Figure S10A, Supporting Information). During adipocyte differentiation, however, the expression of these pro‐osteogenic/anti‐adipogenic transcription factors was not reduced, but even slightly or markedly increased. NPY or/and OCY‐CM did not down‐regulate, but rather up‐regulated the expression of some of these transcription factors, including *Tead1* and *Junb* (Figure 4B; Figure S10B, Supporting Information), which showed the highest levels of inhibition by NPY and OCY‐CM during osteogenic differentiation of BMSCs (Figure 4A). These findings, along with evidence revealing that both NPY and OCY‐CM significantly inhibited the expression of *Tead1* and *Junb* in undifferentiated BMSCs (Figure 4C), suggest that the inhibition of *Tead1* and *Junb* may be critical for the NPY‐ and OCY‐CM‐induced regulation of BMSC fate decision towards adipogenesis rather than osteogenesis, but is not likely to be responsible for further acceleration of differentiation upon adipogenesis. Thus, we overexpressed *Tead1* and *Junb* in undifferentiated BMSCs using recombinant lentiviruses, whose efficacy was verified by qRT‐PCR (Figure 4D) and western blotting (Figure 4E). ARS and ORO staining, respectively, revealed that overexpression of *Tead1* and *Junb* in undifferentiated BMSCs suppressed the anti‐osteogenic and pro‐adipogenic effects of NPY (Figure 4F,G).

NPY can reduce the generation of cyclic adenosine monophosphate (cAMP) to suppress the expression of osteogenic genes in osteoblasts^[^ ^34^ ^]^ and the pro‐osteogenic action of cAMP is mediated by the activation of protein kinase A (PKA) and subsequent the transcription factor cAMP‐response element binding protein (CREB) through the PKA‐mediated CREB phosphorylation.^[^ ^35^ ^]^ Here, ELISA for cellular cAMP revealed that both NPY protein and OCY‐CM could suppress cAMP production in BMSCs under osteogenic or adipogenic induction for 24 h (Figure 4H,I). PKA activity assay and western blotting, respectively, showed the inhibition of PKA activity and CREB phosphorylation in differentiating BMSCs after exposure to NPY protein or OCY‐CM for 24 h (Figure 4J–L). The activation of PKA/CREB signaling by db‐cAMP, a cell‐permeable cAMP analog, reversed the NPY‐induced *Tead1* and *Junb* expression inhibition in BMSCs under osteogenic differentiation (Figure 4M,N). Both the anti‐osteogenic and pro‐adipogenic effects of NPY on BMSCs were profoundly blocked by db‐cAMP (Figure 4O,P). In contrast, blockade of cAMP‐dependent PKA using H‐89 further inhibited *Tead1* and *Junb* expression (Figure 4M,N), reduced osteogenesis, and augmented adipogenesis of the NPY‐treated BMSCs (Figure 4O,P). Together, these results suggest that osteocyte NPY targets *Tead1* and *Junb* by inhibiting cAMP/PKA/CREB signaling to switch BMSC differentiation fate from osteogenesis towards adipogenesis.

It should be noted that cAMP production, PKA activity, and CREB phosphorylation were not only increased during BMSC osteogenesis (Figure 4H,J,L), but also enhanced following adipogenic differentiation of BMSCs (Figure 4I,K,L). The latter might be a compensatory response to counteract excess adipogenesis, which requires future investigation.

We further used chromatin immunoprecipitation (ChIP) assay and qRT‐PCR to determine the binding of transcription factor CREB to the *Tead1* and *Junb* gene promoters and to assess whether NPY can inhibit the binding of CREB to *Tead1* and *Junb*. The results showed that CREB1 antibody, but not the anti‐IgG control, caused a significant enrichment of the promoter regions of *Tead1* and *Junb* relative to the input DNA in the vehicle‐treated BMSCs (Figure 4Q). However, in the NPY‐treated BMSCs, the CREB antibody‐mediated enrichment of either the *Tead1* or *Junb* promoter was markedly impaired (Figure 4Q). These results demonstrate that CREB can bind to the promoter regions of *Tead1* and *Junb* genes, and the NPY‐induced suppression of expression of these two genes in BMSCs is mediated by blocking the binding of CREB to their promoters.

### 2.5 Opposite Modulation of Osteocyte NPY Production and Osteocyte‐Induced Regulation of BMSC Fate by NE and ACh Through *β*2AR and M3R

**Figure 5:**
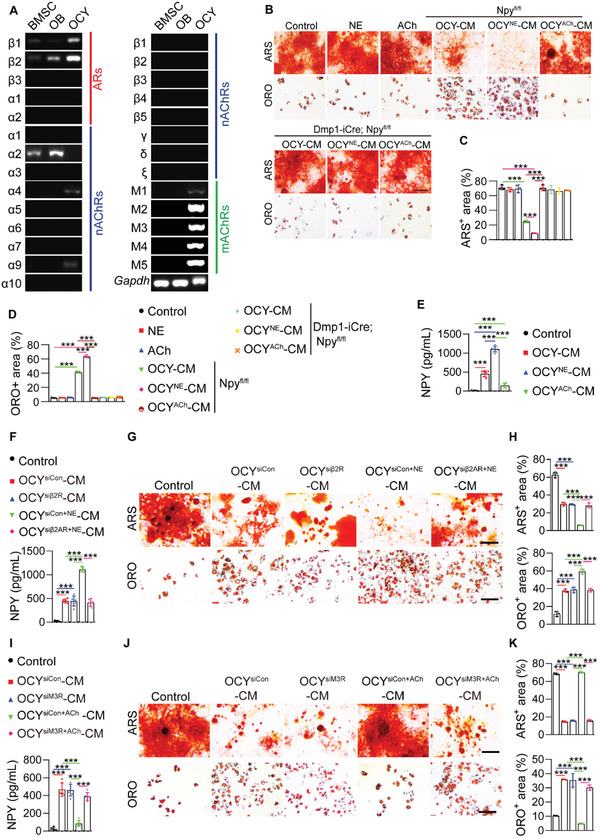
Opposite modulation of osteocyte NPY production and osteocyte‐induced regulation of BMSC fate by NE and ACh through *β*2AR and M3R. A) Semiquantitative PCR analysis of mRNA expression of *α*‐ and *β*‐adrenergic receptors (ARs), muscarinic ACh receptors (mAChRs), and nicotinic (nAChRs) ACh receptors in BMSCs, osteoblasts, and osteocytes from 8‐month‐old male wild‐type mice. B) ARS and ORO staining images of BMSCs with different treatments under osteogenic or adipogenic induction and quantification of the percentages of C) ARS^+^ and D) ORO^+^ areas. Scale bar: 50 µm. *n* = 3 per group. E) ELISA for NPY in un‐cultured medium (Control) and OCY‐CM from osteocytes treated with vehicle (OCY‐CM), NE (OCY^NE^‐CM), or ACh (OCY^ACh^‐CM). *n* = 6 per group. F) ELISA for NPY in un‐cultured medium (Control) and OCY‐CM from osteocytes treated with control siRNAs (OCY^siCon^‐CM), *β*2AR siRNAs (OCY^si*β*2AR^‐CM), siCon + NE (OCY^siCon+NE^‐CM), or si*β*2AR + NE (OCY^si*β*2AR+NE^‐CM). *n* = 6 per group. G) ARS and ORO staining images of BMSCs with different treatments under osteogenic or adipogenic induction and H) the percentages of ARS^+^ and ORO^+^ areas. Scale bar: 50 µm. *n* = 3 per group. I) ELISA for NPY in un‐cultured medium (Control) and OCY‐CM from osteocytes treated with siCon (OCY^siCon^‐CM), M3R siRNAs (OCY^siM3R^‐CM), siCon + ACh (OCY^siCon+ACh^‐CM) or, siM3R + ACh (OCY^siM3R+ACh^‐CM). *n* = 6 per group. J) ARS and ORO staining images of BMSCs receiving different treatments under osteogenic or adipogenic induction and K) the percentages of ARS^+^ and ORO^+^ areas. Scale bar: 50 µm. *n* = 3 per group. Data are presented as mean ± SD. One‐way ANOVA combined with Bonferroni post hoc test. ***P < 0.001.

We next explored which signaling controls osteocyte NPY production and osteocyte‐dependent regulation of BMSC differentiation. Immunofluorescence double staining for DMP1/TH and DMP1/VaChT showed that both TH‐positive sympathetic fibers and VaChT‐positive parasympathetic fibers could be detected in the periosteum, cortical bone, trabecular bone, and bone marrow (Figure S11, Supporting Information), consistent with the evidence in previous studies.^[^ ^18^, ^24^ ^]^ In the mineralized bone, these neuronal fibers were located in the bone matrix and the areas positive for DMP1, which can be secreted into the bone matrix, but can also be detected along the cell membrane, the processes, and nuclei of osteocytes.^[^ ^36^ ^]^ TH^+^/DMP1^+^ and VaChT^+^/DMP1^+^ signals were not only observed in the bone matrix, but were also detected around osteocytes, suggesting that the sympathetic and parasympathetic fibers are able to closely contact with osteocytes. Semiquantitative PCR revealed that osteocytes, but not osteoblasts and BMSCs, predominantly expressed the *β*1 and *β*2 adrenergic receptors (*β*1AR and *β*2AR), the *α*4 and *α*9 nicotinic ACh receptors (*α*4 and *α*9 nAChRs), and five muscarinic ACh receptors (M1‐5 mAChRs) at the mRNA levels (**Figure** **5**A), suggesting that osteocytes are likely the main target of SNS and PSNS for their effects on bone metabolism. As evidenced by ARS and ORO staining, direct treatment with NE and ACh indeed had no obvious effects on osteogenic and adipogenic differentiation of BMSCs in vitro (Figure 5B–D). However, treatment of the *Npy^fl/fl^* mice‐derived normal osteocytes with NE led to further augmented anti‐osteogenic and pro‐adipogenic capacities of their culture media (OCY^NE^‐CM), while ACh caused an opposite effect on the normal osteocytes‐induced regulation of BMSC differentiation (Figure 5B–D). Treatment of the *Dmp1‐iCre*; *Npy^fl/fl^* mice‐derived NPY‐lacking osteocytes with either NE or ACh failed to alter the effects of their culture media on BMSC differentiation (Figure 5B–D). ELISA for OCY‐CM showed that NE profoundly augmented NPY secretion by osteocytes, whereas ACh significantly suppressed osteocyte NPY generation (Figure 5E).

As *β*2AR and M3R have been reported to negatively and positively modulate bone mass, respectively,^[^ ^18^, ^37^ ^]^ and our evidence showed the abundant expression of *β*2AR and M3R mRNAs (*Adrb2* and *Chrm3*) in osteocytes (Figure 5A), we then investigated whether these receptors are involved in the NE‐ and ACh‐induced regulation of osteocyte NPY production and osteocyte‐mediated regulation of BMSC differentiation. ELISA revealed that *β*2AR knockdown by siRNAs had no notable effect on NPY production in the solvent‐treated control osteocytes, but blocked the increase of NPY secretion in the NE‐treated osteocytes (Figure 5F). OCY^si*β*2AR^‐CM exhibited a similar anti‐osteogenic and pro‐adipogenic ability compared with OCY^siCon^‐CM, whereas OCY^si*β*2AR+NE^‐CM failed to further inhibit calcium nodule formation and promote adipogenic differentiation of BMSCs compared to OCY^siCon+NE^ ‐CM (Figure 5G,H). Inhibition of M3R using siRNAs did not affect osteocyte NPY generation and the anti‐osteogenic and pro‐adipogenic capacities of OCY‐CM, but significantly reversed the ACh‐induced suppression of osteocyte NPY production and abrogation of the anti‐osteogenic and pro‐adipogenic effects of OCY‐CM (Figure 5I–K). These findings indicate that NE and ACh, respectively, function through *β*2AR and M3R to oppositely modulate osteocyte NPY production and osteocyte‐dependent regulation of BMSC differentiation.

### 2.6 Osteocyte NPY Mediates the ANS‐Induced Regulation of Bone‐Fat Balance

**Figure 6:**
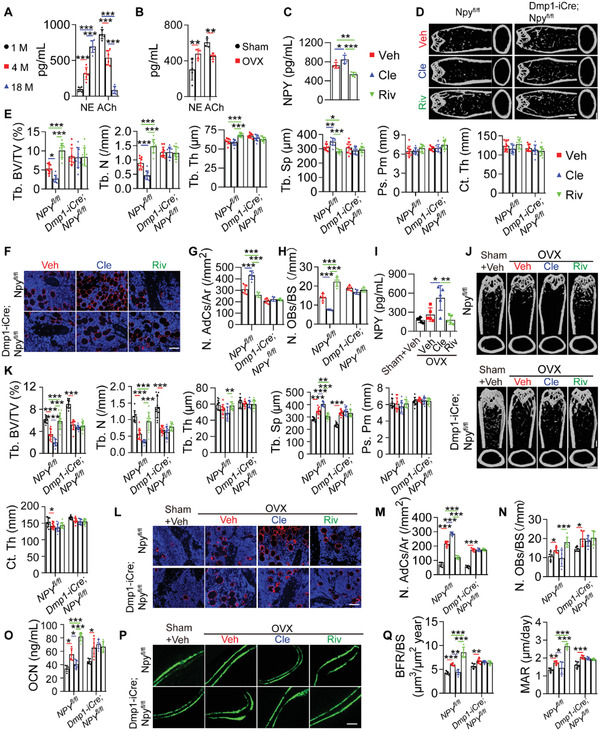
Osteocyte NPY mediates the ANS‐induced regulation of bone‐fat balance. ELISA for NE and ACh in homogenates of the marrow‐depleted femurs A) from normal male mice at different ages (*n* = 6 per group) or B) from sham‐ and OVX‐operated female mice (*n* = 5 per group). C) ELISA for NPY in the marrow‐depleted femur homogenates from 15‐month‐old male *Npy^fl/fl^* mice receiving different treatments for two months. *n* = 5 per group. Cle: clenbuterol; Riv: rivastigmine. D) µCT reconstruction images and E) quantification of bone microarchitecture parameters in femurs from 15‐month‐old male *Npy^fl/fl^* mice and *Dmp1‐iCre*; *Npy^fl/fl^* mice receiving different treatments for two months. Scale bar: 1 mm. *n* = 10 per group. F) Immunofluorescence staining images for perilipin and G) quantification of adipocyte number in distal femurs. Scale bar: 50 µm. *n* = 5 per group. H) Quantification of osteoblast number in distal femurs. *n* = 5 per group. I) ELISA for NPY protein in the marrow‐depleted femur homogenates from 3‐month‐old female *Npy^fl/fl^* mice subjected to sham or OVX operation with different treatments for two months. *n* = 5 per group. J) µCT reconstruction images and K) quantification of bone microarchitecture parameters in femurs from 3‐month‐old female *Npy^fl/fl^* mice and *Dmp1‐iCre*; *Npy^fl/fl^* mice subjected to sham or OVX operation with different treatments for two months. Scale bar: 1 mm. *n* = 10 per group. L) Immunofluorescence staining images for perilipin and M) quantification of adipocyte number in distal femurs. Scale bar: 50 µm. *n* = 5 per group. N) Quantification of osteoblast number in distal femurs. *n* = 5 per group. O) ELISA for serum OCN. *n* = 5 per group. P) Calcein double labeling of trabecular bones and Q) quantification of BFR/BS and MAR. Scale bar: 25 µm. *n* = 5 per group. Data are presented as mean ± SD. For panel (C): one‐way ANOVA combined with Bonferroni post hoc test. For panel (I): unpaired, two‐tailed student's *t*‐test (differences between Sham + Vehicle and OVX + Vehicle groups) or one‐way ANOVA combined with Bonferroni post hoc test (differences among other groups except Sham + Vehicle group). For other dot plots: two‐way ANOVA combined with Bonferroni post hoc test. *P < 0.05, **P < 0.01, and ***P < 0.001.

ELISA revealed the age‐dependent increase of SNS activity and decrease of PSNS activity in the bone tissues by the enhanced level of the sympathetic transmitter NE and the reduced level of the parasympathetic transmitter ACh (**Figure** **6**A). The mice subjected to OVX also showed increased NE and decreased ACh concentrations in the bone tissues compared with the sham‐operated mice (Figure 6B). The above data, along with the opposite effects of NE and ACh on osteocyte NPY generation, suggest that the imbalance of SNS and PSNS activities may be responsible for the aging‐ and OVX‐induced osteocyte NPY overproduction, thus influencing BMSC fate decision and leading to bone‐fat imbalance.

We then tried to assess the involvement of ANS in the regulation of bone‐fat balance and to determine the role of osteocyte NPY in the process. As evidenced by ELISA, subcutaneous injection of the *β*2AR agonist clenbuterol (Cle) to the aged (15 months old) *Npy^fl/fl^* mice for two months caused a significant increase in bone NPY production, while rivastigmine (Riv), a cholinesterase inhibitor that elevates ACh by inhibiting ACh degradation, significantly reduced NPY level in the bone tissues (Figure 6C). NPY protein was almost undetectable in the marrow‐depleted bones from *Dmp1‐iCre*; *Npy^fl/fl^* mice receiving Cle, Riv, or solvent treatments (data not shown). Administration of Cle to *Npy^fl/fl^* mice resulted in a prominent decrease in trabecular bone mass, a trend of decrease in cortical bone mass (Figure 6D,E), significant increase in marrow adipocyte number (Figure 6F,G), and reduction in the number of OCN‐positive osteoblasts (Figure 6H), whereas treatment with Riv generated remarkable bone‐beneficial effects entirely opposite to that of Cle (Figure 6D–H). Neither Cle or Riv induced significant changes in bone mass, marrow adipocyte deposition, and osteoblast number in the osteocyte NPY‐lacking (*Dmp1‐iCre*; *Npy^fl/fl^*) mice (Figure 6D–H). Cle and RiV, respectively, also augmented and reduced NPY levels in the bone tissues from ovariectomized *Npy^fl/fl^* mice (Figure 6I). Bone NPY was barely detectable in *Dmp1‐iCre*; *Npy^fl/fl^* mice treated with either of these agents (data not shown). Similar to that observed in the aged *Npy^fl/fl^* mice, subcutaneous injection of Cle further aggravated the OVX‐induced bone loss (Figure 6J,K) and marrow adiposity (Figure 6L,M), while administration of Riv effectively enhanced trabecular bone mass (Figure 6J,K), decreased the number of marrow adipocytes (Figure 6L,M), and elevated the levels of osteogenic activity (Figure 6N,O) and bone formation/mineralization‐related indicators (Figure 6P,Q) in the ovariectomized *Npy^fl/fl^* mice. The ovariectomized *Dmp1‐iCre*; *Npy^fl/fl^* mice, however, were resistant to the Cle‐ and Riv‐induced regulation of bone mass, marrowfat deposition, osteogenic responses, and new bone formation/mineralization (Figure 6J–Q). Although evidences have shown a stimulatory effect of *β*2AR signaling and an inhibitory action of ACh signaling on osteoclast formation and activity,^[^ ^23^, ^38^ ^]^ here we found that both Cle and Riv did not induce statistically significant changes in the number and activity of osteoclasts in the ovariectomized *Npy^fl/fl^* mice and *Dmp1‐iCre*; *Npy^fl/fl^* mice, as indicated by the number of TRAP^+^ osteoclasts (Figure S12A, Supporting Information) and ELISA for serum CTX‐I (Figure S12B, Supporting Information). Collectively, these results suggest that SNS promotes whereas PSNS attenuates bone‐fat imbalance during skeletal aging and osteoporosis through their opposite regulation of osteocyte NPY production.

### 2.7 *γ*‐Oryzanol Attenuates ANS Dysregulation, NPY Overproduction, and Bone‐Fat Imbalance in Aging‐ and OVX‐Induced Osteoporotic Mice

**Figure 7:**
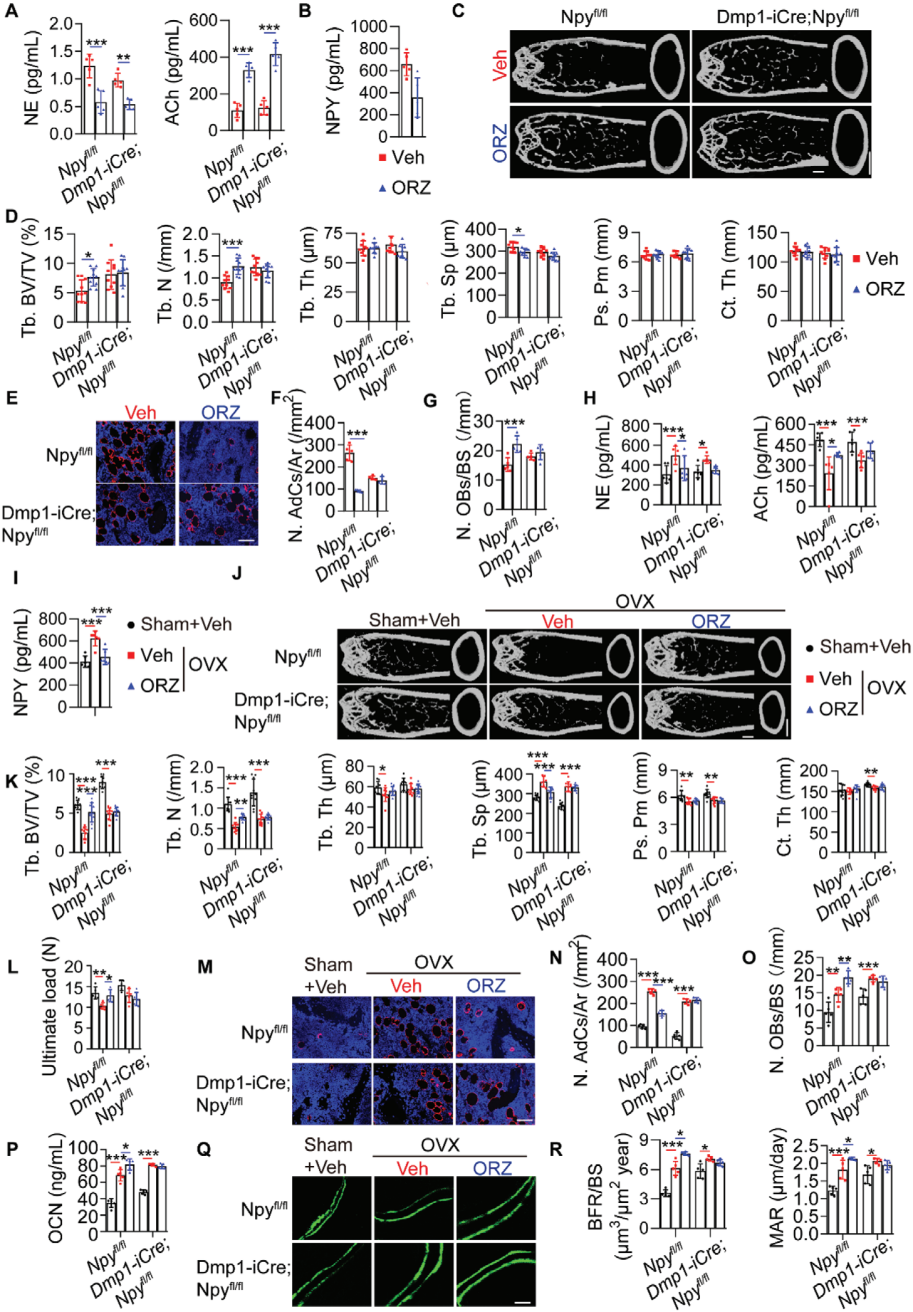
*γ*‐Oryzanol attenuates ANS dysregulation, NPY overproduction, and bone‐fat imbalance in aging‐ and OVX‐induced osteoporotic mice. A) ELISA for NE and ACh in homogenates of the marrow‐depleted femurs from 15‐month‐old male *Npy^fl/fl^* mice and *Dmp1‐iCre*; *Npy^fl/fl^* mice with solvent or *γ*‐Oryzanol treatments for two months. ORZ: *γ*‐Oryzanol*. n* = 5 per group. B) ELISA for NPY in homogenates of the marrow‐depleted femurs from 15‐month‐old male *Npy^fl/fl^* mice in different treatment groups. *n* = 5 per group. C) µCT reconstruction images and D) quantification of bone microarchitecture parameters in femurs. Scale bar: 1 mm. *n* = 10 per group. E) Immunofluorescence staining images for perilipin in distal femurs and F) quantification of adipocyte number. Scale bar: 50 µm. *n* = 5 per group. G) Quantification of osteoblast number in distal femurs. *n* = 5 per group. H) ELISA for NE and ACh in homogenates of the marrow‐depleted femurs from 3‐month‐old female *Npy^fl/fl^* mice and *Dmp1‐iCre*; *Npy^fl/fl^* mice subjected to sham or OVX operation with solvent or *γ*‐Oryzanol treatments for two months. *n* = 6 per group. I) ELISA for NPY in homogenates of the marrow‐depleted femurs from female *Npy^fl/fl^* mice in different treatment groups. *n* = 6 per group. J) µCT reconstruction images and K) quantification of bone microarchitecture parameters in femurs. Scale bar: 1 mm. *n* = 10 per group. L) Three‐point bending measurement of femur ultimate load. *n* = 5 per group. M) Immunofluorescence staining images for perilipin in distal femurs and N) quantification of adipocyte number. Scale bar: 50 µm. *n* = 5 per group. O) The number of osteoblasts in distal femurs. *n* = 5 per group. P) ELISA for serum OCN. *n* = 5 per group. Q) Calcein double labeling of trabecular bones and R) quantification of BFR/BS and MAR. Scale bar: 25 µm. *n* = 5 per group. Data are presented as mean ± SD. For panel (B) and (I): unpaired, two tailed student's *t*‐test. For other dot plots: two‐way ANOVA combined with Bonferroni post hoc test. *P < 0.05, **P < 0.01, and ***P < 0.001.

Finally, we investigated the impact of *γ*‐Oryzanol on osteocyte NPY production and bone‐fat imbalance during aging and estrogen deficiency‐induced osteoporosis. Using ELISA for homogenates of the bone marrow‐depleted femurs, we found that daily intragastric administration of *γ*‐Oryzanol for two months markedly reduced the level of NE and enhanced the level of ACh in the bone tissues from aged (15 months old) *Npy^fl/fl^* mice and *Dmp1‐iCre*; *Npy^fl/fl^* mice (**Figure** **7**A). A prominent decrease of bone NPY production was observed in *Npy^fl/fl^* mice after *γ*‐Oryzanol treatment (Figure 7B), whereas NPY in the marrow‐depleted bone was almost undetectable in *Dmp1‐iCre*; *Npy^fl/fl^* mice treated with solvent or *γ*‐Oryzanol (data not shown). Similar to that observed in the Riv‐treated mice, *γ*‐Oryzanol administration profoundly enhanced trabecular bone mass, diminished marrow fat accumulation, and increased osteoblast numbers in the aged *Npy^fl/fl^* mice, but not in the aged *Dmp1‐iCre*; *Npy^fl/fl^* mice, as revealed by µCT analysis (Figure 7C,D), immunofluorescence staining for perilipin (Figure 7E), and quantitative analyses of the number of marrow adipocytes (Figure 7F) and OCN^+^ osteoblasts (Figure 7G), respectively. *γ*‐Oryzanol treatment also resulted in profound inhibitions of the OVX‐induced increase of NE, a decrease of ACh, and enhancement of NPY protein in the bone tissues of *Npy^fl/fl^* mice (Figure 7H,I). The ovariectomized *Npy^fl/fl^* mice treated with *γ*‐Oryzanol had significantly less bone loss (Figure 7J,K) with stronger bone strength (Figure 7L), lower level of bone marrow adipocyte accumulation (Figures 7M,N), higher osteoblast number and activity (Figure 7O,P), and greater capacity to form new bone (Figure 7Q,R), as compared with the vehicle‐treated *Npy^fl/fl^* OVX mice. However, even if *γ*‐Oryzanol could also markedly reduce the level of NE and enhanced the level of ACh in the bone tissues from the ovariectomized *Dmp1‐iCre*; *Npy^fl/fl^* mice (Figure 7H), it had lost the ability to generate significant bone‐protective effects in these osteocyte NPY‐lacking mice (Figure 7J–R). TRAP staining and ELISA for serum CTX‐I showed that the number and activity of osteoclasts were not notably altered after *γ*‐Oryzanol treatment in the ovariectomized *Npy^fl/fl^* mice and *Dmp1‐iCre*; *Npy^fl/fl^* mice (Figure S12C,S12D, Supporting Information). These results reveal the therapeutic benefits of the ANS regulator *γ*‐Oryzanol as an osteocyte NPY‐targeting agent against aging‐ and OVX‐induced bone loss and marrow adiposity.

**Figure 8:**
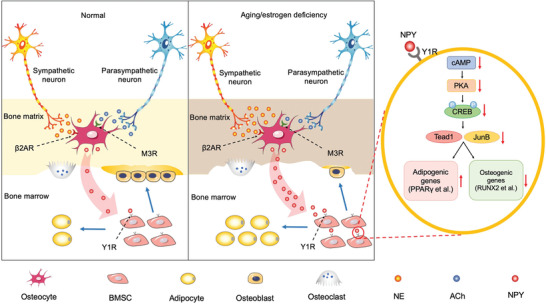
Schematic diagram showing an ANS‐osteocyte NPY‐mediated mechanism by which aging and estrogen deficiency switch BMSC differentiation fate from osteogenesis towards adipogenesis and induce bone loss and marrow adiposity.

Taken together, the data in our study present a new mode of neuronal control of bone‐fat balance through the ANS‐induced modulation of osteocyte NPY (**Figure** **8**). The sympathetic and parasympathetic nerve terminals in bone release NE and ACh, respectively, to stimulate and inhibit the production of NPY through their respective receptors (*β*2AR and M3R) in osteocytes. Osteocyte NPY acts through Y1R to suppress the expression of pro‐osteogenic/anti‐adipogenic transcription factors *Tead1* and *Junb* by inhibiting cAMP/PKA/CREB signaling, thus switching BMSC fate from osteogenesis towards adipogenesis. In young and estrogen‐normal body, the production of the sympathetic transmitter NE and parasympathetic transmitter ACh is maintained at a balanced level in bone, which makes osteocytes cannot generate enough NPY to favor BMSC differentiation towards adipogenesis. With aging or estrogen deficiency due to menopause, the increase of SNS activity and decrease of PSNS activity lead to the overproduction of NE and reduction of ACh, which result in excess osteocyte NPY generation and subsequent bone‐fat imbalance.

## 3 Discussion

DMP1 is an extracellular matrix protein highly expressed in osteocytes within the bone matrix and in osteocyte precursors (preosteocytes or late/mature osteoblasts) that are located near the BS but partially embedded in the bone matrix.^[^ ^16^, ^30^, ^39^ ^]^ DMP1 is also detected in a few osteoblasts at embryonal stage, but less expressed by osteoblasts in postnatal life.^[^ ^16^, ^30^, ^39^ ^]^ As osteocytes are the most abundant cells in bone and abundantly express DMP1 at embryonal and post‐natal stages, *Dmp1*‐*Cre* mice have been widely used in osteocyte‐specific deletion.^[^ ^9^, ^11^, ^25^, ^40^ ^]^ Nevertheless, *Dmp1‐Cre* can inevitably target a minority of osteoblasts that express DMP1. Mature osteoblasts and bone lining cells (derived from mature osteoblasts and located on the BS)^[^ ^41^ ^]^ will also be targeted by *Dmp1‐Cre* due to their DMP1 expression. In our study, we did not find and use a perfect Cre model that only targets osteocytes. Although our results showed that NPY protein was absent in osteocytes, but was still present in many osteoblasts of *Dmp1‐iCre*; *Npy^fl/fl^* mice, we could not rule out the contribution of *Dmp1*‐expressing osteoblasts to the observed bone phenotype in *Dmp1‐iCre*; *Npy^fl/fl^* mice. We could not also rule out the contribution of mature osteoblasts and their descendant bone lining cells to the bone phenotype in *Dmp1‐iCre*; *Npy^fl/fl^* mice. However, the bone phenotype in *Dmp1‐iCre*; *Npy^fl/fl^* mice could not be attributed equally to osteocytes and osteoblasts. Osteocytes are the most abundant (>90%) bone cells and the major cells that express DMP1 in bone.^[^ ^5^ ^]^ Both our results and the data provided by other study^[^ ^16^ ^]^ demonstrated that osteocytes, but not osteoblasts, were the major cell source for NPY. These findings, along with the results showing the absence of NPY in osteocytes and the presence of NPY in many osteoblasts in *Dmp1‐iCre*; *Npy^fl/fl^* mice, indicate that osteocytes are the major bone cells that contribute to the bone phenotype in *Dmp1‐iCre*; *Npy^fl/fl^* mice.

In addition to osteocytes/preosteocytes and a few osteoblasts, DMP1 expression is also found in the tooth germ and non‐mineralized tissues including brain, pancreas, and kidney.^[^ ^42^ ^]^ Consistently, we also identified the presence of DMP1 protein in the mouse tooth germ with the surrounding bone, islets of the pancreas, renal tubules, and renal cortex, but NPY protein was rarely detected in these sites (data not shown). In the brain, DMP1 protein showed a nuclear‐ or nuclear outside‐location in the NPY protein‐absent cerebral cortex and pyramidal cell layer of the hippocampus, with little expression in the NPY protein‐abundant hypothalamus. Few DMP1/NPY‐double‐positive cells in the tooth germ, pancreas, kidney, and brain suggest that *Dmp1‐iCre* may not induce a significant effect on NPY expression in these tissues. The results of western blotting and immunostaining for NPY in the present study indicate that NPY expression in the NPY‐abundant brain including the area of the hypothalamus is not notably affected in *Dmp1‐iCre*; *Npy^fl/fl^* mice. No significant changes of body weight, appetite, and metabolic indicators between *Dmp1‐iCre*; *Npy^fl/fl^* mice and *Npy^fl/fl^* mice also suggest that hypothalamic NPY is not profoundly influenced in *Dmp1‐iCre*; *Npy^fl/fl^* mice, since hypothalamic NPY is a potent orexigenic factor that can promote energy storage.^[^ ^28^ ^]^ Our findings suggest that the higher bone mass phenotypes in *Dmp1‐iCre*; *Npy^fl/fl^* mice are mainly mediated by locally targeting osteocyte NPY. Nevertheless, we just provided a lot of indirect evidence to rule out the contribution of hypothalamic NPY to the bone phenotype in *Dmp1‐iCre*; *Npy^fl/fl^* mice. Future studies are required to cross *Dmp1‐iCre* mice to a reporter line (such as Ai14 mice) to carefully check the deletion pattern of *Dmp1‐iCre* in the hypothalamus, in order to directly rule out the hypothalamic NPY contribution to the observed bone phenotype in *Dmp1‐iCre*; *Npy^fl/fl^* mice.

The evidence of the existence of neuronal regulation of bone homeostasis starts with the discovery that leptin, a hormone primarily secreted by adipocytes, inhibits bone formation through the SNS‐induced inhibition of osteoblast proliferation and function.^[^ ^21^, ^43^ ^]^ As a powerful orexigenic factor opposite to leptin, NPY does not antagonize leptin in the control of bone formation, but also functions as an inhibitor of osteogenic activity in the CNS and periphery.^[^ ^43^ ^]^ In our study, we further demonstrated a local regulatory role of NPY in bone via the Y1R‐mediated inhibition of osteogenesis and promotion of adipogenesis of BMSCs and showed the existence of autonomic control of bone NPY production and bone‐fat balance in skeletal aging and osteoporosis. We identified osteocytes as the major source of NPY in bone to temporally produce NPY under the control of ANS and spatially affect BMSC fate decision and subsequent bone‐fat balance by inhibiting the pro‐osteogenic/anti‐adipogenic transcription factors TEAD1 and JUNB through cAMP/PKA/CREB signaling. Osteocyte NPY was required for the SNS‐induced promotion and PSNS‐induced inhibition of bone‐fat imbalance and osteoporosis. Specific deletion of NPY in osteocytes or increase of PSNS signaling by injection of rivastigmine, significantly enhanced bone formation and ameliorated aging‐ and OVX‐associated bone loss and marrow adipocyte accumulation.

Osteocytes form a neuron‐like network through their long dendritic processes to communicate with neighboring osteocytes and with cells on the BS such as osteoblasts and osteoclasts.^[^ ^5^, ^6^ ^]^ The osteocyte network can sense mechanical loading and convert them to chemical signals.^[^ ^44^ ^]^ It may also easily and efficiently sense and respond to chemical signals from other cells. In our study, we found that the sympathetic transmitter NE and the parasympathetic transmitter ACh could function through their respective receptors (*β*2AR and M3R) on osteocytes to respectively stimulate and inhibit osteocyte NPY production and then alter the ability of osteocytes to modulate BMSC differentiation fate. Thus, even though the vast majority of osteocytes were far away from the sympathetic and parasympathetic nerves, the transmitters NE and ACh could be released from the nerves to the bone matrix upon SNS and PSNS activation, respectively, as our results showed that these transmitters could be detected in the homogenates of the marrow‐depleted femurs. These transmitters then could act through *β*2AR and M3R on osteocytes to innervate osteocytes and modulate NPY production. For the trabecular bone, although the sympathetic and parasympathetic nerves were mainly surrounding but not inside the bone, the transmitters could still be released from the nerves surrounding the trabecular bone and then diffused or transported to the bone matrix and the osteocyte network. However, it remains to be further investigated how these transmitters enter into the receptors on osteocyte membrane.

During osteoclastic bone resorption, growth factors deposited in the bone matrix can be released into the bone marrow cavity to regulate bone homeostasis.^[^ ^45^ ^]^ In our study, using an in vitro model, we demonstrated that incubation with osteoclasts resulted in a significant increase of NPY release from the bone slices into the culture medium. This result, along with the evidences that bone resorption activity is relatively or absolutely augmented during senile or postmenopausal osteoporosis^[^ ^46^, ^47^ ^]^ and the contributory role of osteocyte NPY in aging‐ and OVX‐induced bone‐fat imbalance determined in our study, suggests that osteocyte NPY can be released from the bone matrix to the bone marrow cavity during skeletal aging and menopause, which finally switches BMSC differentiation fate and induces bone‐fat imbalance.

Rice bran is a by‐product of brown rice milling and demonstrated to be a valuable source of bioactive and nutritional compounds.^[^ ^48^ ^]^ *γ*‐Oryzanol, a mixture of ferulic acid esters with triterpene alcohols, is an important component of rice bran that has various health‐beneficial effects (hypolipidemic, anti‐inflammatory, anti‐diabetic, etc.)^[^ ^48^, ^49^ ^]^ and has been used for the cure of the syndromes of ANS imbalance and climacteric disturbance.^[^ ^27^ ^]^ Studies have shown that oral treatment with *γ*‐Oryzanol effectively enhances the expression of osteogenesis‐related genes and bone mass in OVX mice.^[^ ^50^ ^]^ However, evidence is lacking on the impact of *γ*‐Oryzanol on bone‐fat imbalance and the underlying molecular mechanism by which *γ*‐Oryzanol modulates bone metabolism. Here we determined that daily intake of *γ*‐Oryzanol for two months was sufficient to prevent aging‐ and OVX‐induced ANS dysregulation, osteocyte NPY production, and bone‐fat imbalance. Our study demonstrates an osteocyte NPY‐dependent neuronal control of BMSC fate decision and bone‐fat balance, and suggests that diet‐ or drug‐based therapy that favors ANS balance and reduces osteocyte NPY production is a promising therapeutic option against osteoporosis and skeletal aging.

## 4 Experimental Section

### Mice and Treatments

The experimental protocol in this study was approved by the Ethics Committee of Xiangya Hospital of Central South University. Female and male C57BL/6 wild‐type mice, *Dmp1‐iCre* mice, *Npy^fl/−^* mice*, Npy^fl/fl^* mice, *Dmp1‐iCre*; *Npy^fl/fl^* mice, *Col1a1‐CreERT2* mice, and *Col1a1‐Cre/ERT2*; *Npy^fl/fl^* mice at different ages were used for animal experiments. C57BL/6 wild‐type mice were purchased from Hunan SJA Laboratory Animal Co., Ltd (Changsha, China). *Dmp1‐iCre* mice were kindly gifted from Prof. Yao Sun (Tongji University). *Col1a1‐CreERT2* mice were obtained from Jackson Laboratory (Stock No: 01 6241).

*Npy^fl/fl^* mice and *Dmp1‐iCre* mice were generated using gene‐targeting techniques in C57BL/6 mouse embryonic stem (ES) cells by Cyagen Biosciences Inc. (Guangzhou, China) and Biocytogen Co., Ltd (Beijing, China), respectively. To generate *Npy* floxed allele, a loxP site was introduced to intron 1 and an SDA (self‐deletion anchor)‐neo (neomycin resistance gene)‐SDA‐loxP cassette was introduced to intron 2 of *Npy* gene, which eventually formed a targeting construct containing a loxP‐exon 2‐SDA‐neo‐SDA‐loxP allele. The targeting construct was used to electroporate ES cells and the neomycin‐resistant ES cells were then identified and injected into C57BL/6 albino embryos, which were re‐implanted into pseudo‐pregnant females to generate chimeric mice. After cross‐breeding with the C57BL/6 mice, the neomycin resistance gene was automatically deleted in their offspring. After several generations of breeding, their germline transmission was confirmed by genotyping of the offspring using their genomic DNA extracted from tail tips. The primers designed to confirm the insertion of the loxP linker into the exact restriction site with correct direction were shown as below: loxP‐F (F1): 5’‐TCCCAGACGCCAGTGAACTTGC‐3’; loxP‐R (R1): 5’‐AGGTGTTCCCAGGTTCTTCTCCC‐3’. The primers R1 and R2 will amplify a product of 262 bp if the loxP linker was inserted, or 206 bp in the case of no insertion. The following primers were used for detecting the deletion of neomycin‐resistant gene: Neo‐del‐F (F2): 5’‐CAGGGGTGCAGAAGTGTAAAGGT‐3’; Neo‐del‐R (R2): 5’‐CAGAGTCCCTAATGTAAAGGCTTTG‐3’. Wild‐type allele generated a band at 314 bp, while the floxed allele generated a product of 427 bp. To generate *Dmp1‐iCre* mice, the *iCre* gene with a viral 2A sequence (*2A*‐*iCre*) was introduced between exon 6 and 3’ UTR in the mouse *Dmp1* gene*. Dmp1* and *iCre* could be expressed at the same time and at the same level with the use of the 2A expression system. The neo cassette flanked by two FRT sites was inserted within the non‐conserved region of exon 5 of *Dmp1* gene. The targeting vector was electroporated into C57BL/6 ES cells and the targeted ES cells carrying the *Dmp1‐2A*‐*iCre* allele were injected into blastocysts to produce chimeric mice. The neo cassette was deleted by breeding with the transgenic mice expressing Flp recombinase. *Dmp1‐iCre* mice were crossed with the *Npy* floxed heterozygous (*Npy^fl/−^*) or homozygous (*Npy^fl/fl^*) mice. Their offspring were intercrossed to generate *Dmp1‐iCre*; *Npy^fl/fl^* mice. Genotyping for *iCre* was conducted by PCR with the primers as follows: Dmp1‐iCre‐FRT‐F: 5’‐CACGTCCTCTCACTTCTCACG‐3’; Dmp1‐iCre‐FRT‐R: 5’‐CTTTGACAGTGTCTTATCCAATAGCC‐3’. The primers amplify a 341‐bp product in mice hemizygous for the *Dmp1‐iCre* allele and a 240‐bp product in wild‐type mice.

3‐week‐old wild‐type mice were used for harvesting BMSCs to compare the effects of different treatments on osteogenesis and adipogenesis of BMSCs. 4‐month‐old male wild‐type mice were used for detecting NPY expression profiles in different tissues and for assessing the distribution of TH‐ or VaChT‐positive neuronal fibers in the bone tissues. 1‐, 4‐, and 18‐month‐old male wild‐type mice were used for testing the effects of OCY‐CM on BMSC differentiation, the expression changes of NPY in femurs, brain, and osteocytes, and the concentrations of NE and ACh in the bone tissues. 8–10‐month‐old male wild‐type mice were used for testing NPY expression in bone‐related cells and for isolating osteocytes to assess the role NPY or/and its downstream factors in osteocytes‐induced regulation of BMSC differentiation. 3‐month‐old female C57BL/6 wild‐type mice were subjected to bilateral OVX or Sham surgery as described in the previous studies.^[^ ^51^, ^52^ ^]^ NPY expression in their femurs, brain, and osteocytes was detected by western blotting after operation for 15 days. OCY‐CM was obtained from their osteocytes for examining the levels of NPY protein and the effects of OCY‐CM on BMSC differentiation. The levels of NE and ACh in the bone tissues from these mice were assessed.

Bone marrow‐depleted or non‐depleted femurs, whole bone marrow cells, brain, hypothalamus, and bone‐related cells were obtained from 4‐month‐old male *Npy^fl/fl^* mice and *Dmp1‐iCre*; *Npy^fl/fl^* mice for confirming the osteocyte‐specific deletion of *Npy* in *Dmp1‐iCre*; *Npy^fl/fl^* mice. Bone mass and microstructure in 3‐month‐old male *Dmp1‐iCre* mice, *Npy^fl/fl^* mice, *Dmp1‐iCre*; *Npy^fl/fl^* mice, and their wild‐type littermates were compared. 3‐ and 18‐month‐old male, and 3‐month‐old female *Npy^fl/fl^* mice and *Dmp1‐iCre*; *Npy^fl/fl^* mice were used for evaluating the role of osteocyte NPY in aging‐ and OVX‐induced bone loss and marrow adiposity. Body weights of male *Dmp1‐iCre*; *Npy^fl/fl^* mice and *Npy^fl/fl^* mice at the age of 1 to 5 months were recorded. Daily intake of food and water, lean mass, fat mass, and blood metabolic indicators were measured in 3‐month‐old female *Dmp1‐iCre*; *Npy^fl/fl^* mice and *Npy^fl/fl^* mice. 8–10‐month‐old gender‐matched *Npy^fl/fl^* mice and *Dmp1‐iCre*; *Npy^fl/fl^* mice were used for isolating osteocytes to compare the effects of their OCY‐CM on BMSC differentiation. BMSCs from 3‐week‐old gender‐matched *Npy^fl/fl^* mice and *Dmp1‐iCre*; *Npy^fl/fl^* mice were harvested for comparing their osteogenic and adipogenic differentiation abilities. To explore the impact of SNS and PSNS activation and *γ*‐Oryzanol on bone‐fat balance and the role of osteocyte NPY in this process, 3‐month‐old female *Npy^fl/fl^* mice and *Dmp1‐iCre*; *Npy^fl/fl^* mice subjected to OVX and 15‐month‐old male *Npy^fl/fl^* mice and *Dmp1‐iCre*; *Npy^fl/fl^* mice were subcutaneously injected with Cle (1 mg kg^−1^/day; MedChemExpress, Monmouth Junction, NJ, USA), Riv (1 mg kg^−1^/day; MedChemExpress), or vehicle (PBS), or treated with *γ*‐Oryzanol (Orz; 100 mg kg^−1^/day; MedChemExpress) or vehicle (Corn oil) by intragastric administration. Mice in the Sham group were daily treated with an equal volume of vehicle. 2 months later, the mice were killed and the serum samples were obtained as previously described.^[^ ^1^, ^47^ ^]^ The femora were harvested and processed for further analyses.

To assess the effect of NPY deletion in osteoblasts on bone mass, *Col1a1‐CreERT2* mice were crossed with *Npy^fl/fl^* mice and their offspring were then intercrossed to produce *Col1a1‐Cre/ERT2*; *Npy^fl/fl^* mice. Primers for detecting the presence of Cre sites were displayed as follows: Cre‐F: 5’‐TCCAATTTACTGACCGTACACCAA‐3’; Cre‐R: 5’‐CCTGATCCTGGCAATTTCGGCTA‐3’. The primers will amplify a product of 500‐bp. Male *Col1a1‐Cre/ERT2*; *Npy^fl/fl^* mice and *Npy^fl/fl^* mice at the age of 3 months were intraperitoneally injected with tamoxifen (75mg kg^−1^; Sigma‐Aldrich, St. Louis, MO, USA) in corn oil (Sigma‐Aldrich) every day for 5 days and then left for two months. The mice were then killed to obtain femurs for µCT analysis.

### µCT Analysis

After being fixed for 2 days with 4% paraformaldehyde (PFA), the right femurs were scanned by vivaCT80 (SCANCO Medical AG, Bruettisellen, Switzerland), with a resolution of 11.4 μm per pixel, a voltage of 55 kV, and a current of 145 μA. Images were reconstructed using NRecon software and visualized using μCTVol v2.2 software. The areas between 0.45 and 0.90 mm proximal to the growth plate of the distal femurs were selected for trabecular bone analysis, in order to evaluate Tb. BV/TV, Tb. N, Tb. Th, and Tb. Sp. The regions for cortical bone analysis were 5% of the femoral length in the femoral mid‐diaphysis, in order to assess Ps. Pm and Ct.Th.

### Three‐Point Bending Test

Bone strength was assessed by the three‐point bending test with a mechanical testing machine from Shanghai Zhuoji Instruments Co. Ltd. (WD‐D1; Shanghai, China) as previously described.^[^ ^47^ ^]^ There are a central loading point and two fulcrums with a distance of 8 mm in the femurs. The specimens were loaded at a constant speed of 5 mm min^−1^ until failure and the ultimate load value (N) was calculated from the load‐deformation curves.

### Histological and Immunohistochemical Staining

The 4% PFA‐fixed, 18% EDTA‐decalcified, and paraffin‐embedded left femur samples were prepared and sectioned into 5 µm‐thick slices. Immunofluorescence staining for perilipin was conducted to test the changes of bone marrow adipocytes. Perilipin antibody was purchased from Sigma‐Aldrich. Osteogenic and osteoclastic activities, respectively, were evaluated by immunohistochemical staining for OCN using antibodies from Servicebio (Wuhan, China) and TRAP staining using a kit from Sigma‐Aldrich. Adipocytes, OCN^+^ osteoblasts, and TRAP^+^ osteoclasts were counted from four random visual fields of distal metaphysis for each femur section and five mouse femur samples for each group. The numbers of adipocytes per area (N. AdCs/Ar/mm^2^) and the numbers of osteoblasts (N. OBs/BS/mm) and osteoclasts (N. OCs/BS/mm) per BS were calculated. Immunofluorescence double staining for DMP1/NPY and immunohistochemical staining for NPY were conducted to assess DMP1 and NPY expression in the bone tissues. Immunofluorescence double staining for DMP1/TH and DMP1/VaChT was performed to evaluate the ANS innervation of osteocytes. Anti‐NPY and anti‐TH were obtained from Cell Signaling Technology (Danvers, USA). Anti‐DMP1 and anti‐VaChT were purchased from Novus Biologicals (Littleton, USA) and Sigma‐Aldrich, respectively.

### Histomorphometric Analysis

For double calcein labeling, mice in different groups were injected intraperitoneally with 0.1% calcein (10 mg kg^−1^; Sigma‐Aldrich) at 10 days and 3 days before euthanasia. The femurs were obtained and fixed for 2 days with 4% PFA. After dehydration, the methyl methacrylate‐embedded specimens were prepared and sectioned into 150 µm‐thick slices, which were polished to a final thickness of about 40 µm. Calcein double labeling was detected using a Zeiss fluorescence microscope (Jena, Germany). Bone dynamic histomorphometric analyses for BFR/BS and MAR of trabecular bone were conducted with the Image‐Pro Plus 6 software.

### Cell Isolation, Culture, and Characterization

Primary osteocytes and osteoblasts were isolated from the mouse femurs and tibias using the method utilizing sequential collagenase and EDTA treatments. The procedures were described in detail in the study published by Stern AR et al.^[^ ^53^ ^]^ Briefly, the femurs and tibias were dissected from soft tissue and placed in *α*‐minimal essential medium (*α*‐MEM; Hyclone, Logan, USA) containing 10% Penicillin‐Streptomycin (PS; Solarbio, Beijing, China). After removal of the remaining muscle and connective tissue, the periosteum was scraped off and the bones were washed using *α*‐MEM with 10% PS, followed by cutting away the epiphyses. The bones were flushed with *α*‐MEM containing 10% PS to remove bone marrow and then cut into bone pieces with lengths of about 1 mm in Hank's balanced salt solution (HBSS). Bone pieces were incubated three times (25 min each time) with collagenase type IA (300 U mL^−1^; Sigma‐Aldrich) in *α*‐MEM. The solution was aspirated for cell plating. Every time after collagenase digestion, the bone pieces were washed with HBSS and the HBSS rinse was added to the aspirated solution for cell plating. Cells in the solution were pelleted, resuspended, and seeded in the collagen‐coated plates with *α*‐MEM containing 5% fetal bovine serum (FBS; Gibco, Grand Island, USA), 5% fetal calf serum (FCS; Gibco), and 1% PS. Cells obtained after collagenase digestion and HBSS wash for two or three times would be primarily osteoblasts. The bone pieces were then incubated for 25 min with EDTA solution (5 mm in magnesium‐and calcium‐free Dulbecco's PBS containing 1% BSA), washed with HBSS, digested for 25 min with 300 U mL^−1^ collagenase type IA, and then washed again with HBSS. The solution aspirated after collagenase digestion and HBSS wash was centrifuged at 200 ×g for 5 min and subjected to cell plating. Cells in the solution would be also primarily osteoblasts. After another two times EDTA–HBSS–collagenase–HBSS treatments, the osteocyte‐enriched fractions could be obtained. The remaining bone pieces were minced using a tissue homogenizer in *α*‐MEM. The osteocyte‐enriched fractions and the resulting bone particle suspension were then plated in the collagen‐coated plates and cultured in *α*‐MEM with 5% FBS, 5% FCS, and 1% PS. Primary monocytes/macrophages were harvested from the mouse bone marrow by flushing the femurs and tibias using the method described in detail in the previous study.^[^ ^52^ ^]^ Upon treatment with M‐CSF (30 ng mL^−1^; R&D Systems Inc., Minneapolis, USA) and RANKL (100 ng mL^−1^; PeproTech, Rocky Hill, USA) in *α*‐MEM containing 10% FBS and 1% PS for 8 days, these cells could differentiate and fuse into multinucleated osteoclasts. Primary BMSCs were isolated from the bone marrow of the mouse femurs and tibias according to the protocol reported by Zhu and colleagues.^[^ ^54^ ^]^ BMSCs were cultured in *α*‐MEM with 10% FBS and 1% PS. RAW264.7 cells (ATCC, Rockville, USA) were incubated in high glucose DMEM (Gibco) containing 10% FBS and 1% PS. Cells were maintained in a 5% CO_2_ incubator at 37 °C.

Cell morphology was observed with an optical microscope. Immunofluorescence staining for DMP1 and ALP staining was conducted to identify osteocytes. Osteoblasts were characterized by immunofluorescence staining for OCN and ALP staining. The expression of Sca‐1, CD44, CD90, CD45, and CD34 on BMSCs was detected using flow cytometry. The antibodies were obtained from BD Biosciences (San Jose, USA). Osteogenic and adipogenic differentiation capacities of BMSCs were evaluated by culturing these cells in osteogenic medium (Cyagen Biosciences, Guangzhou, China) or adipogenic medium (Cyagen) for 8 days. The formation of mineralized nodules and lipid droplets, respectively, was evaluated by ARS and ORO staining using the kits from Solarbio. Monocytes/macrophages were identified by F4/80 and CD11b double staining with flow cytometry. Osteoclasts were detected by TRAP staining with a kit from Sigma‐Aldrich.

### OCY‐CM Preparation

The culture medium of osteocytes (OCY‐CM) was harvested and centrifuged for 10 min at 300 ×g and for 30 min at 2000 ×g to remove dead cells and debris. 4 mL supernatant was concentrated to 100 to 200 µL by centrifugation at 4000 ×g with an Amicon Ultra‐4 Centrifugal Filter Unit (3 kDa; Millipore, Billerica, USA). The concentrated OCY‐CM was obtained and the protein concentration of OCY‐CM was measured with a BCA Protein Assay Kit (Thermo Fisher Scientific, USA). OCY‐CM samples were used for the downstream assays or stored at −80 °C.

### Osteogenic and Adipogenic Differentiation Assays

BMSCs at passage 1 or 2 were seeded into 48‐well plates at a density of 1 × 10^5^ cells per well for osteogenic induction and 2 × 10^5^ cells per well for adipogenic induction. 24 h later, the culture medium was replaced with fresh osteogenic or adipogenic medium (Cyagen) supplemented with OCY‐CM (300 µg mL^−1^ at the protein level) from different groups, recombinant NPY protein (0.1 nm; MedChemExpress), BIBO3304 (0.1 nm; Selleck Chemicals, Houston, USA), NPY (0.1 nm) + db‐cAMP (10 µm; MedChemExpress), NPY (0.1 nm) + H‐89 (10 µm; MedChemExpress), NE (0.1 nm; Sigma‐Aldrich), ACh (0.1 nm; Sigma‐Aldrich), or an equal volume of vehicle. The induction medium was replaced with fresh osteogenic or adipogenic medium containing the corresponding supplements every other day. Cells cultured in *α*‐MEM containing 10% FBS and 1% PS served as negative controls. In order to detect the changes of cAMP production, PKA activity, and CREB phosphorylation, the cells subjected to induction for 24 h were processed for ELISA for cellular cAMP, PKA activity assay, and western blotting for CREB and phosphorylated CREB. After differentiation for 3 days, total RNA of the differentiated BMSCs was extracted and the expression of a class of pro‐osteogenic/anti‐adipogenic transcription factors or genes related to osteogenesis or adipogenesis was assessed by qRT‐PCR. After differentiation for 8 days, the cells were stained with ARS solution (Solarbio) to evaluate matrix mineralization, or stained with ORO solution (Solarbio) to detect lipid droplet formation. The percentages of ARS^+^ and ORO^+^ areas were analyzed from five random visual fields for each biological replicate and three biological replicates for each group.

### Osteoclastic Differentiation Assay

RAW264.7 cells (1 × 10^4^ cells per well) were incubated in 48‐well plates for 24 h. After that, the culture medium was replaced with fresh complete DMEM supplemented with RANKL (100 ng mL^−1^) or RANKL (100 ng mL^−1^) + NPY (0.1 nm; MedChemExpress). The medium was changed every other day. After differentiation for 8 days, the cells were stained with TRAP reagents to detect the formation of osteoclasts, which showed more than three nuclei. The numbers of osteoclasts were counted from five random visual fields for each biological replicate and three biological replicates for each group.

### Preparation of Bone‐Resorption CM

RAW264.7 cells were cultured in an osteoclastic induction medium (complete DMEM + 100 ng mL^−1^ RANKL) and several osteoclasts were formed after induction for 4 days. Bone slices prepared from the periosteum‐ and bone marrow‐depleted femurs and tibias of 4‐month‐old wild‐type mice were equally placed into the cultures of the differentiating RAW264.7 cells. The induction medium was changed every other day. At 8 days after induction, the medium was replaced with a fresh serum‐free osteoclastic induction medium. After incubation for another 48 h, the bone‐resorption CM was obtained for ELISA to test the concentration of NPY protein. Osteoclastic induction medium only, bone slices incubated in osteoclastic induction medium without cells, and RAW264.7 cells cultured in osteoclastic induction medium without bone slices served as controls.

### ELISA

The concentrations of OCN and CTX‐I in serum, NPY protein in OCY‐CM, bone resorption CM, and marrow‐depleted bone homogenates, as well as NE and ACh in marrow‐depleted bone homogenates were tested using ELISA kits purchased from Elabscience (Wuhan, China).

### PKA Activity Assay

The culture media of differentiated BMSCs in different groups were incubated with the reagents from a PKA kinase assay kit (ImmuneChem, Burnaby, Canada) and the absorbance was assessed at 450 nm using a Varioskan LUX Multimode microplate reader (Thermo Fisher Scientific).

### RNA Interference or Gene Overexpression

Three NPY siRNAs (siNPY #1, 2, and 3), *β*2AR siRNAs (si*β*2AR #1, 2, and 3), and M3R siRNAs (siM3R #1, 2, and 3) were purchased from RiboBio (Guangzhou, China) and employed to interfere with the expression of NPY, *β*2AR, and M3R in osteocytes, respectively. The control cells were transfected with the universal negative control siRNAs (siCon). After 24 h of transfection, the cells were processed for qRT‐PCR and the siRNAs with the highest inhibitory efficiency were selected for further assays. The siRNA sequences were as follows: siNPY #1: 5’‐CGACACTACATCAATCTCA‐3’; siNPY #2: 5’‐CTTGAAGA CCCTTCCATGT‐3’; siNPY #3: 5’‐CCCTGAGACACTGATTTCA‐3’; si*β*2AR #1: 5’‐CCATCCTCATGTCGGTTAT‐3’; si*β*2AR #2: 5’‐GCTGCAGAAGATAGACAAA‐3’; si*β*2AR #3: 5’‐GGAAGGAACTGTAGTACAA‐3’; siM3R #1: 5’‐GGCAGTTCTCGAAGCTGTA‐3’; siM3R #2: 5’‐CTATGGATGTAGAGAGAAA‐3’; siM3R #3: 5’‐CCAGAAGTCAGATCACCAA‐3’. For overexpression of TEAD1 and JUNB in BMSCs, the cloned *Tead1* or *Junb* gene ORF was inserted into the lentiviral vector pLV[Exp]‐EGFP:T2A:Puro‐EF1A with a FLAG‐tag (YunZhou Biotech Co. Ltd., Guangzhou, China). Cell transfection and puromycin selection were conducted according to the handbook from the manufacturer. Cells in the control group were transfected with the lentiviruses carrying an empty vector. The overexpression efficiency was confirmed by qRT‐PCR and western blotting.

### Semiquantitative PCR and qRT‐PCR

Total RNA of cells was extracted and then reversely transcribed to cDNA with the All‐in‐One cDNA Synthesis SuperMix (Biotool, Houston, USA). The products were subjected to semiquantitative analysis using PCR followed by agarose gel electrophoresis, or quantitative analysis by qRT‐PCR, which was conducted on the FTC‐3000 real‐time PCR system (Funglyn Biotech Inc, Toronto, Canada). GAPDH or *β*‐actin served as the internal control. Primers were listed in Table S1, Supporting Information.

### Western Blotting

Western blotting was conducted as described previously.^[^ ^1^ ^]^ Briefly, an equal amount of protein extracts (30 µg) were subjected to SDS‐PAGE (15% gel for NPY and 12% gel for other antibodies) and transferred to PVDF membranes, followed by blocking for 1 h in 5% milk and incubation overnight at 4 °C with anti‐NPY (1:1000; Santa Cruz Biotechnology, Santa Cruz, USA), anti‐NPY1R (1:1000; Zen Bioscience, Chengdu, China), anti‐TEAD1 (1:1000; Servicebio), anti‐JUNB (1:1000; Zen Bioscience), anti‐CREB (1:1000; Servicebio), anti‐p‐CREB (1:1000; Zen Bioscience), or anti‐*β*‐actin (1:1000; Servicebio). After that, the membranes were washed and incubated at room temperature for 1 h with the secondary antibodies (1:5000; Servicebio). After washing, protein bands were examined by an enhanced chemiluminescence kit from Advansta (Menlo Park, USA).

### ChIP Assay

ChIP assay was performed using a commercial kit from Cell Signaling Technology. Briefly, BMSCs treated with vehicle or NPY protein (0.1 nm) for 24 h were fixed at room temperature with 1% formaldehyde for 10 min, quenched for 5 min with glycine solution, washed with ice‐cold PBS, resuspended in buffers supplied with the kit, and then digested at 37 °C with micrococcal nuclease for 20 min. The chromatin fragments were obtained and then immunoprecipitated with the ChIP‐validated antibodies against CREB (1:50; Cell Signaling Technology), normal rabbit IgG (negative control antibody), or histone H3 antibody overnight at 4 °C, followed by incubation at 4 °C with ChIP‐Grade Protein G Magnetic Beads for 2 h. After washing, the eluted DNA was purified and assessed by qRT‐PCR to measure the amount of enrichment of the region of the *Tead1* or *Junb* gene promoter. Histone H3 antibody pulldown for the enrichment of ribosomal protein L30 (*Rpl30*) gene served as a positive control. Primers used for qRT‐PCR were shown as follows: mouse‐*Tead1*: forward, 5’‐ CCTGGAATTTGCTATATAAACCTCA‐3’, and reverse, 5’‐TATTCCTTCTAGAGAGGTGATGTGG‐3’; mouse‐*Junb*: forward, 5’‐TCTTCTCAGGAAGATCTCAGATAC C‐3’, and reverse, 5’‐AATTTTGGGGAGGTCAATTTAGATA‐3’. *Rpl30* primers were provided with the kit.

### Statistical Analysis

Data were presented as mean ± SD. The unpaired, two‐tailed student's *t*‐test was used for comparing mean differences between two groups. One‐way analysis of variance (ANOVA) or two‐way ANOVA combined with Bonferroni post hoc test were adopted for multiple‐group comparisons. A difference with *P* < 0.05 was considered statistically significant. GraphPad Prism 8 software was used for statistical analyses. The sample size (n) for each statistical analysis was detailed in each figure legend.

## Conflict of Interest

The authors declare no conflict of interest.

## Author Contributions

Y.Z. and C.‐Y.C. contributed equally to this work. H.X. and C.Y.C. conceived the research. Y.Z., Y.W.L., S.S.R., Y.J.T., Y.X.Q., K.X., X.X.L, C.G.H., H.Y., J.H., J.C., S.K.F., Z.H.H., Y.Y.L., Z.W.L., B.W., Z.Q.Y., T.H.C., M.L.C., Y.Y.W., Z.X.W., L.J., and T.F.W performed the experiments. Y.Z., C.Y.C., S.S.R., H.Y., and J.H. analyzed the data and prepared figures. Z.Z.L., M.J.L., X.K.H., T.Y., and S.Y.T provided technical support. H.X. and C.Y. C. wrote the manuscript.

## Supporting information

Supporting Information

## Data Availability

Research data are not shared.

## References

[1] S. S. Rao , Y. Hu , P. L. Xie , J. Cao , Z. X. Wang , J. H. Liu , H. Yin , J. Huang , Y. J. Tan , J. Luo , M. J. Luo , S. Y. Tang , T. H. Chen , L. Q. Yuan , E. Y. Liao , R. Xu , Z. Z. Liu , C. Y. Chen , H. Xie , Bone Res. 2018, 6, 9. 29619269 10.1038/s41413-018-0012-0 PMC5876344

[2] T. D. Rachner , S. Khosla , L. C. Hofbauer , Lancet 2011, 377, 1276. 21450337 10.1016/S0140-6736(10)62349-5 PMC3555696

[3] B. Yu , L. Huo , Y. Liu , P. Deng , J. Szymanski , J. Li , X. Luo , C. Hong , J. Lin , C. Y. Wang , Cell Stem Cell 2018, 23, 615. 30244868 10.1016/j.stem.2018.09.001 PMC6613582

[4] a) Y. Fan , J. I. Hanai , P. T. Le , R. Bi , D. Maridas , V. DeMambro , C. A. Figueroa , S. Kir , X. Zhou , M. Mannstadt , R. Baron , R. T. Bronson , M. C. Horowitz , J. Y. Wu , J. P. Bilezikian , D. W. Dempster , C. J. Rosen , B. Lanske , Cell Metab. 2017, 25, 661; 28162969 10.1016/j.cmet.2017.01.001 PMC5342925

[5] S. Tatsumi , K. Ishii , N. Amizuka , M. Li , T. Kobayashi , K. Kohno , M. Ito , S. Takeshita , K. Ikeda , Cell Metab. 2007, 5, 464. 17550781 10.1016/j.cmet.2007.05.001

[6] J. Gao , A. Qin , D. Liu , R. Ruan , Q. Wang , J. Yuan , T. S. Cheng , A. Filipovska , J. M. Papadimitriou , K. Dai , Q. Jiang , X. Gao , J. Q. Feng , H. Takayanagi , C. Zhang , M. H. Zheng , Sci. Adv. 2019, 5, eaaw7215. 31799389 10.1126/sciadv.aaw7215 PMC6867880

[7] a) Y. Uda , E. Azab , N. Sun , C. Shi , P. D. Pajevic , Curr. Osteoporos. Rep. 2017, 15, 318; 28612339 10.1007/s11914-017-0373-0 PMC5656287

[8] a) W. Liu , Z. Wang , J. Yang , Y. Wang , K. Li , B. Huang , B. Yan , T. Wang , M. Li , Z. Zou , J. Yang , G. Xiao , Z. K. Cui , A. Liu , X. Bai , Open Biol. 2019, 9, 180262; 31088250 10.1098/rsob.180262 PMC6544986

[9] K. S. Joeng , Y. C. Lee , J. Lim , Y. Chen , M. M. Jiang , E. Munivez , C. Ambrose , B. H. Lee , J. Clin. Invest. 2017, 127, 2678. 28628032 10.1172/JCI92617 PMC5490765

[10] a) P. Divieti Pajevic , D. S. Krause , Bone 2019, 119, 13; 29458123 10.1016/j.bone.2018.02.012 PMC6095825

[11] J. Xiong , M. Onal , R. L. Jilka , R. S. Weinstein , S. C. Manolagas , C. A. O'Brien , Nat. Med. 2011, 17, 1235. 21909103 10.1038/nm.2448 PMC3192296

[12] a) J. M. Brazill , A. T. Beeve , C. S. Craft , J. J. Ivanusic , E. L. Scheller , J. Bone Miner. Res. 2019, 34, 1393; 31247122 10.1002/jbmr.3822 PMC6697229

[13] a) P. A. Baldock , N. J. Lee , F. Driessler , S. Lin , S. Allison , B. Stehrer , E. J. Lin , L. Zhang , R. F. Enriquez , I. P. Wong , M. M. McDonald , M. During , D. D. Pierroz , K. Slack , Y. C. Shi , E. Yulyaningsih , A. Aljanova , D. G. Little , S. L. Ferrari , A. Sainsbury , J. A. Eisman , H. Herzog , PLoS One 2009, 4, e8415; 20027231 10.1371/journal.pone.0008415 PMC2794533

[14] J. M. Lundberg , A. Martinsson , A. Hemsen , E. Theodorsson‐Norheim , J. Svedenhag , B. Ekblom , P. Hjemdahl , Biochem. Biophys. Res. Commun. 1985, 133, 30. 3840999 10.1016/0006-291x(85)91837-6

[15] J. M. Lundberg , T. Hokfelt , A. Hemsen , E. Theodorsson‐Norheim , J. Pernow , B. Hamberger , M. Goldstein , Regul. Pept. 1986, 13, 169. 3513267 10.1016/0167-0115(86)90224-7

[16] J. C. Igwe , X. Jiang , F. Paic , L. Ma , D. J. Adams , P. A. Baldock , C. C. Pilbeam , I. Kalajzic , J. Cell. Biochem. 2009, 108, 621. 19670271 10.1002/jcb.22294 PMC2754602

[17] a) N. J. Lee , K. L. Doyle , A. Sainsbury , R. F. Enriquez , Y. J. Hort , S. J. Riepler , P. A. Baldock , H. Herzog , J. Bone Miner. Res. 2010, 25, 1736; 20200977 10.1002/jbmr.61

[18] F. Elefteriou , Physiol. Rev. 2018, 98, 1083. 29717928 10.1152/physrev.00014.2017 PMC6088147

[19] a) P. Balasubramanian , D. Hall , M. Subramanian , Geroscience 2019, 41, 13; 30519806 10.1007/s11357-018-0048-5 PMC6423215

[20] A. Togari , M. Arai , A. Kondo , Expert Opin. Ther. Targets 2005, 9, 931. 16185149 10.1517/14728222.9.5.931

[21] S. Takeda , F. Elefteriou , R. Levasseur , X. Liu , L. Zhao , K. L. Parker , D. Armstrong , P. Ducy , G. Karsenty , Cell 2002, 111, 305. 12419242 10.1016/s0092-8674(02)01049-8

[22] A. Tosun , M. T. Dogru , G. Aydn , I. Keles , A. Arslan , M. Guneri , S. Orkun , H. Ebinc , Am. J. Phys. Med. Rehabil. 2011, 90, 1012. 22019965 10.1097/PHM.0b013e31822dea1a

[23] H. Eimar , S. Alebrahim , G. Manickam , A. Al‐Subaie , L. Abu‐Nada , M. Murshed , F. Tamimi , Bone 2016, 84, 131. 26719214 10.1016/j.bone.2015.12.009

[24] a) D. B. Mach , S. D. Rogers , M. C. Sabino , N. M. Luger , M. J. Schwei , J. D. Pomonis , C. P. Keyser , D. R. Clohisy , D. J. Adams , P. O'Leary , P. W. Mantyh , Neuroscience 2002, 113, 155; 12123694 10.1016/s0306-4522(02)00165-3

[25] N. Asada , Y. Katayama , M. Sato , K. Minagawa , K. Wakahashi , H. Kawano , Y. Kawano , A. Sada , K. Ikeda , T. Matsui , M. Tanimoto , Cell Stem Cell 2013, 12, 737. 23746979 10.1016/j.stem.2013.05.001

[26] Y. Zhu , Y. Ma , F. Elefteriou , Bone Rep. 2018, 9, 188. 30581894 10.1016/j.bonr.2018.11.002 PMC6296164

[27] a) K. Itaya , J. Kiyonaga , Nippon Yakurigaku Zasshi 1976, 72, 475; 987976 10.1254/fpj.72.475

[28] a) C. K. Ip , L. Zhang , A. Farzi , Y. Qi , I. Clarke , F. Reed , Y. C. Shi , R. Enriquez , C. Dayas , B. Graham , D. Begg , J. C. Bruning , N. J. Lee , D. Hernandez‐Sanchez , G. Gopalasingam , J. Koller , R. Tasan , G. Sperk , H. Herzog , Cell Metab. 2019, 30, 111; 31031093 10.1016/j.cmet.2019.04.001

[29] L. E. Kuo , J. B. Kitlinska , J. U. Tilan , L. Li , S. B. Baker , M. D. Johnson , E. W. Lee , M. S. Burnett , S. T. Fricke , R. Kvetnansky , H. Herzog , Z. Zukowska , Nat. Med. 2007, 13, 803. 17603492 10.1038/nm1611

[30] N. Kamiya , M. Takagi , Histochem. J. 2001, 33, 545. 12005026 10.1023/a:1014955925339

[31] M. Okamoto , J. Murai , Y. Imai , D. Ikegami , N. Kamiya , S. Kato , Y. Mishina , H. Yoshikawa , N. Tsumaki , J. Bone Miner. Res. 2011, 26, 2511. 21786321 10.1002/jbmr.477

[32] A. Rauch , A. K. Haakonsson , J. G. S. Madsen , M. Larsen , I. Forss , M. R. Madsen , E. L. Van Hauwaert , C. Wiwie , N. Z. Jespersen , M. Tencerova , R. Nielsen , B. D. Larsen , R. Rottger , J. Baumbach , C. Scheele , M. Kassem , S. Mandrup , Nat. Genet. 2019, 51, 716. 30833796 10.1038/s41588-019-0359-1

[33] J. H. Hong , E. S. Hwang , M. T. McManus , A. Amsterdam , Y. Tian , R. Kalmukova , E. Mueller , T. Benjamin , B. M. Spiegelman , P. A. Sharp , N. Hopkins , M. B. Yaffe , Science 2005, 309, 1074. 16099986 10.1126/science.1110955

[34] H. Horsnell , P. A. Baldock , Curr. Osteoporos. Rep. 2016, 14, 26. 26872458 10.1007/s11914-016-0300-9

[35] R. Siddappa , A. Martens , J. Doorn , A. Leusink , C. Olivo , R. Licht , L. van Rijn , C. Gaspar , R. Fodde , F. Janssen , C. van Blitterswijk , J. de Boer , Proc. Natl. Acad. Sci. U. S. A. 2008, 105, 7281. 18490653 10.1073/pnas.0711190105 PMC2387183

[36] I. Maciejewska , D. Qin , B. Huang , Y. Sun , G. Mues , K. Svoboda , L. Bonewald , W. T. Butler , J. Q. Feng , C. Qin , Cells Tissues Organs 2009, 189, 186. 18698129 10.1159/000151372 PMC2727859

[37] a) N. Bonnet , C. L. Benhamou , B. Brunet‐Imbault , A. Arlettaz , M. N. Horcajada , O. Richard , L. Vico , K. Collomp , D. Courteix , Bone 2005, 37, 622; 16157516 10.1016/j.bone.2005.07.012

[38] a) S. J. Aitken , E. Landao‐Bassonga , S. H. Ralston , A. I. Idris , Arch. Biochem. Biophys. 2009, 482, 96; 19059194 10.1016/j.abb.2008.11.012

[39] a) I. Kalajzic , A. Braut , D. Guo , X. Jiang , M. S. Kronenberg , M. Mina , M. A. Harris , S. E. Harris , D. W. Rowe , Bone 2004, 35, 74; 15207743 10.1016/j.bone.2004.03.006

[40] Y. Lu , Y. Xie , S. Zhang , V. Dusevich , L. F. Bonewald , J. Q. Feng , J. Dent. Res. 2007, 86, 320. 17384025 10.1177/154405910708600404

[41] a) T. Mizoguchi , N. Ono , J. Bone Miner. Res. 2021, 36, 1432; 34213032 10.1002/jbmr.4410 PMC8338797

[42] a) M. Terasawa , R. Shimokawa , T. Terashima , K. Ohya , Y. Takagi , H. Shimokawa , J. Bone Miner. Metab. 2004, 22, 430; 15316863 10.1007/s00774-004-0504-4

[43] P. Ducy , M. Amling , S. Takeda , M. Priemel , A. F. Schilling , F. T. Beil , J. Shen , C. Vinson , J. M. Rueger , G. Karsenty , Cell 2000, 100, 197. 10660043 10.1016/s0092-8674(00)81558-5

[44] A. G. Robling , L. F. Bonewald , Annu. Rev. Physiol. 2020, 82, 485. 32040934 10.1146/annurev-physiol-021119-034332 PMC8274561

[45] a) J. L. Crane , X. Cao , J. Mol. Med. 2014, 92, 107; 24068256 10.1007/s00109-013-1084-3 PMC3946714

[46] Y. Hu , R. Xu , C. Y. Chen , S. S. Rao , K. Xia , J. Huang , H. Yin , Z. X. Wang , J. Cao , Z. Z. Liu , Y. J. Tan , J. Luo , H. Xie , Metabolism 2019, 95, 93. 30668962 10.1016/j.metabol.2019.01.009

[47] C. Y. Chen , S. S. Rao , Y. J. Tan , M. J. Luo , X. K. Hu , H. Yin , J. Huang , Y. Hu , Z. W. Luo , Z. Z. Liu , Z. X. Wang , J. Cao , Y. W. Liu , H. M. Li , Y. Chen , W. Du , J. H. Liu , Y. Zhang , T. H. Chen , H. M. Liu , B. Wu , T. Yue , Y. Y. Wang , K. Xia , P. F. Lei , S. Y. Tang , H. Xie , Bone Res. 2019, 7, 18. 31263627 10.1038/s41413-019-0056-9 PMC6594995

[48] a) H. Masuzaki , C. Kozuka , S. Okamoto , M. Yonamine , H. Tanaka , M. Shimabukuro , J. Diabetes Invest. 2019, 10, 18; 10.1111/jdi.12892 PMC6319487 29978570

[49] O. Wang , J. Liu , Q. Cheng , X. Guo , Y. Wang , L. Zhao , F. Zhou , B. Ji , PLoS One 2015, 10, e0118135. 25646799 10.1371/journal.pone.0118135 PMC4315454

[50] a) S. I. Muhammad , I. Maznah , R. B. Mahmud , M. F. Esmaile , A. B. Zuki , Clin. Interventions Aging 2013, 8, 1421; 10.2147/CIA.S49704 PMC3810202 24187491

[51] J. Huang , H. Yin , S. S. Rao , P. L. Xie , X. Cao , T. Rao , S. Y. Liu , Z. X. Wang , J. Cao , Y. Hu , Y. Zhang , J. Luo , Y. J. Tan , Z. Z. Liu , B. Wu , X. K. Hu , T. H. Chen , C. Y. Chen , H. Xie , Theranostics 2018, 8, 2435. 29721090 10.7150/thno.22144 PMC5928900

[52] H. Xie , Z. Cui , L. Wang , Z. Xia , Y. Hu , L. Xian , C. Li , L. Xie , J. Crane , M. Wan , G. Zhen , Q. Bian , B. Yu , W. Chang , T. Qiu , M. Pickarski , L. T. Duong , J. J. Windle , X. Luo , E. Liao , X. Cao , Nat. Med. 2014, 20, 1270. 25282358 10.1038/nm.3668 PMC4224644

[53] A. R. Stern , L. F. Bonewald , Methods Mol. Biol. 2015, 1226, 3. 25331038 10.1007/978-1-4939-1619-1_1

[54] H. Zhu , Z. K. Guo , X. X. Jiang , H. Li , X. Y. Wang , H. Y. Yao , Y. Zhang , N. Mao , Nat. Protoc. 2010, 5, 550. 20203670 10.1038/nprot.2009.238

