# Supplementary material for: Neuronal Induction of Bone‐Fat Imbalance through Osteocyte Neuropeptide Y
Source: Adv Sci (Weinh). 2021 Oct 31;8(24):2100808. doi: 10.1002/advs.202100808 (PMC8693044; doi:10.1002/advs.202100808)
Supplement: Supplementary file 1 — Supporting Information [file ADVS-8-2100808-s001.pdf]

© 2021 Wiley-VCH GmbH

ADVANCED SCIENCE
Open Access

Supporting Information

for *Adv. Sci.,* DOI: 10.1002/advs.202100808

Neuronal induction of bone-fat imbalance through osteocyte neuropeptide Y

*Yan Zhang, Chun-Yuan Chen, Yi-Wei Liu, Shan-Shan Rao, Yi-Juan Tan, Yu-Xuan Qian, Kun Xia, Jie Huang, Xi-Xi Liu, Chun-Gu Hong, Hao Yin, Jia Cao, Shi-Kai Feng, Ze-Hui He, You-You Li, Zhong-Wei Luo, Ben Wu, Zi-Qi Yan, Tuan-Hui Chen, Meng-Lu Chen, Yi-Yi Wang, Zhen-Xing Wang, Zheng-Zhao Liu, Ming-Jie Luo, Xiong-Ke Hu, Ling Jin, Teng-Fei Wan, Tao Yue, Si-Yuan Tang, Hui Xie***

Supplemental information

Neuronal induction of bone-fat imbalance through osteocyte neuropeptide Y

Yan Zhang[#], Chun-Yuan Chen[#], Yi-Wei Liu, Shan-Shan Rao, Yi-Juan Tan, Yu-Xuan Qian，Kun Xia, Jie Huang，Xi-Xi Liu, Chun-Gu Hong, Hao Yin, Jia Cao, Shi-Kai Feng, Ze-Hui He, You-You Li, Zhong-Wei Luo, Ben Wu, Zi-Qi Yan, Tuan-Hui Chen, Meng-Lu Chen, Yi-Yi Wang, Zhen-Xing Wang, Zheng-Zhao Liu, Ming-Jie Luo, Xiong-Ke Hu, Ling Jin, Teng-Fei Wan, Tao Yue, Si-Yuan Tang, Hui Xie[*]

[#] Yan Zhang and Chun-Yuan Chen contributed equally to this work.

[*] Corresponding author: Hui Xie (huixie@csu.edu.cn).

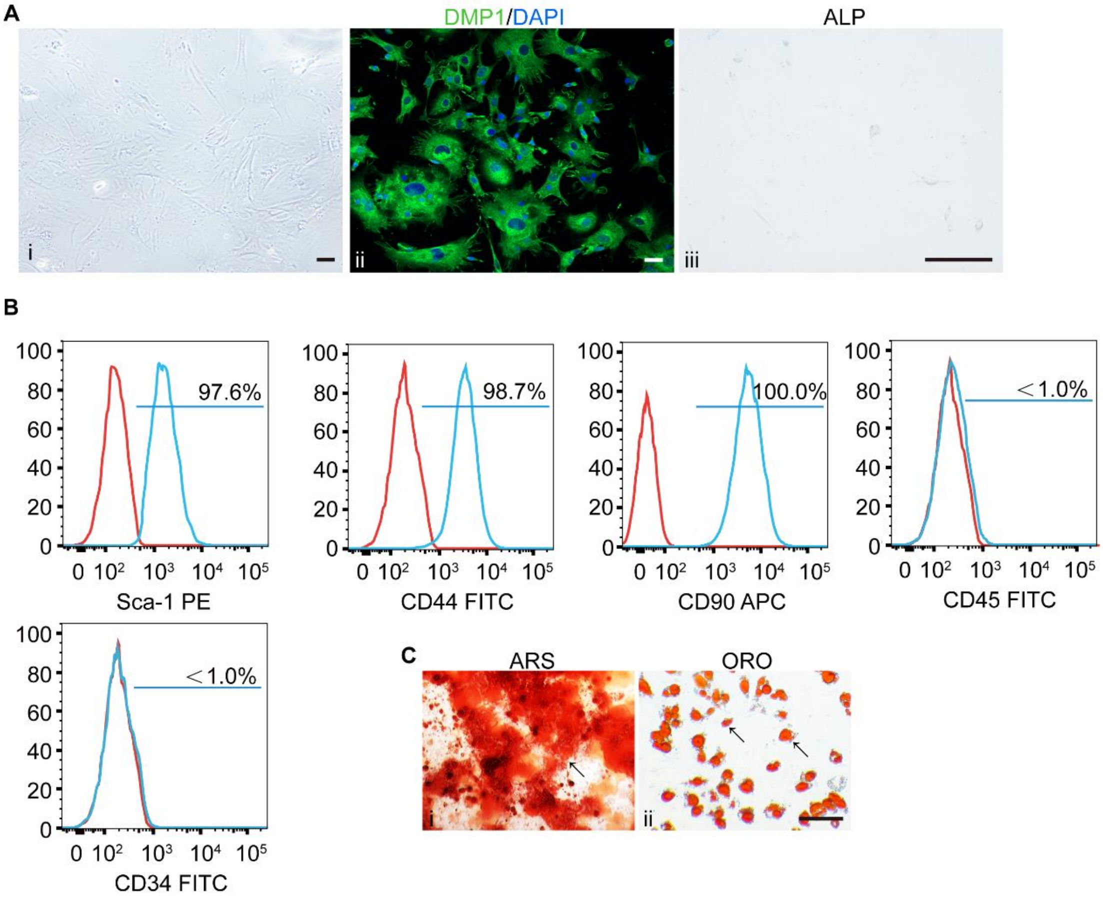

Figure S1. Characterization of osteocytes and BMSCs. A) Osteocytes exhibited a stellate morphology with long dendritic processes under an optical microscope (**i**; Scale bar: 100 μm), expressed DMP1 protein (**ii**; Scale bar: 50 μm), but had no ability to produce ALP protein (**iii**; Scale bar: 100 μm). **B**) Flow cytometry analysis of expression of cell surface markers on BMSCs. The test samples were illustrated as blue curves and the isotype controls were illustrated as red curves. **C**) BMSCs could differentiate into osteoblasts and adipocytes, as detected by ARS (**i**) and ORO (**ii**) staining, respectively. Black arrows indicate the ARS-stained mineralized nodules and ORO-stained lipid droplets. Scale bar: 50 μm.

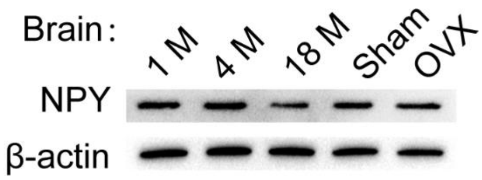

Figure S2. Protein expression of brain NPY. Western blotting for NPY protein in the brain tissues from normal male mice at different ages or female mice subjected to OVX or sham operation.

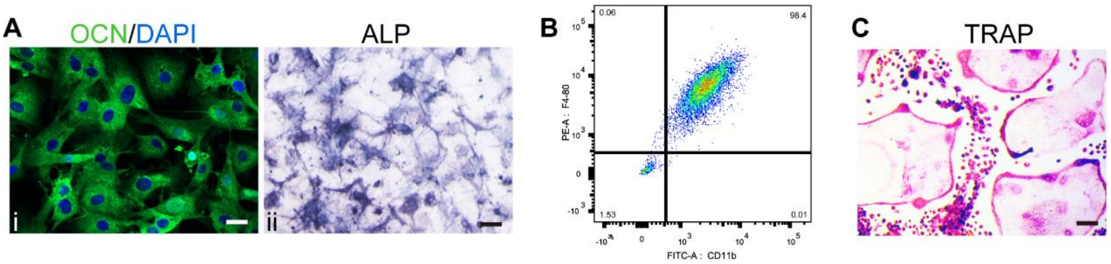

Figure S3. Identification of osteoblasts, monocytes/macrophages, and osteoclasts. **A**) Immunofluorescence staining for OCN (**i**; Scale bar: 50 μm) and ALP staining (**ii**; Scale bar: 100 μm) in osteoblasts. **B**) Flow cytometry analysis of expression of F4/80 and CD11b in monocytes/macrophages. **C**) TRAP staining of osteoclasts formed by monocytes/macrophages treated with osteoclastic induction medium for 8 days. Scale bar: 100 μm.

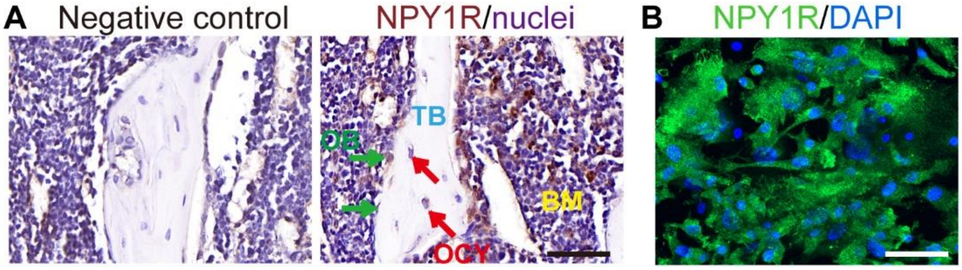

Figure S4. Protein expression of Y1R in the femurs and primary BMSCs. A) Immunohistochemical staining for Y1R in the femur section from 4-month-old male wild-type mice. Secondary antibody only served as negative control. OCY: osteocyte; OB: osteoblast; TB: trabecular bone; BM: bone marrow. Scale bar: 50 μm. **B**) Immunofluorescence staining for Y1R in the cultured primary BMSCs. Scale bar: 50 μm.

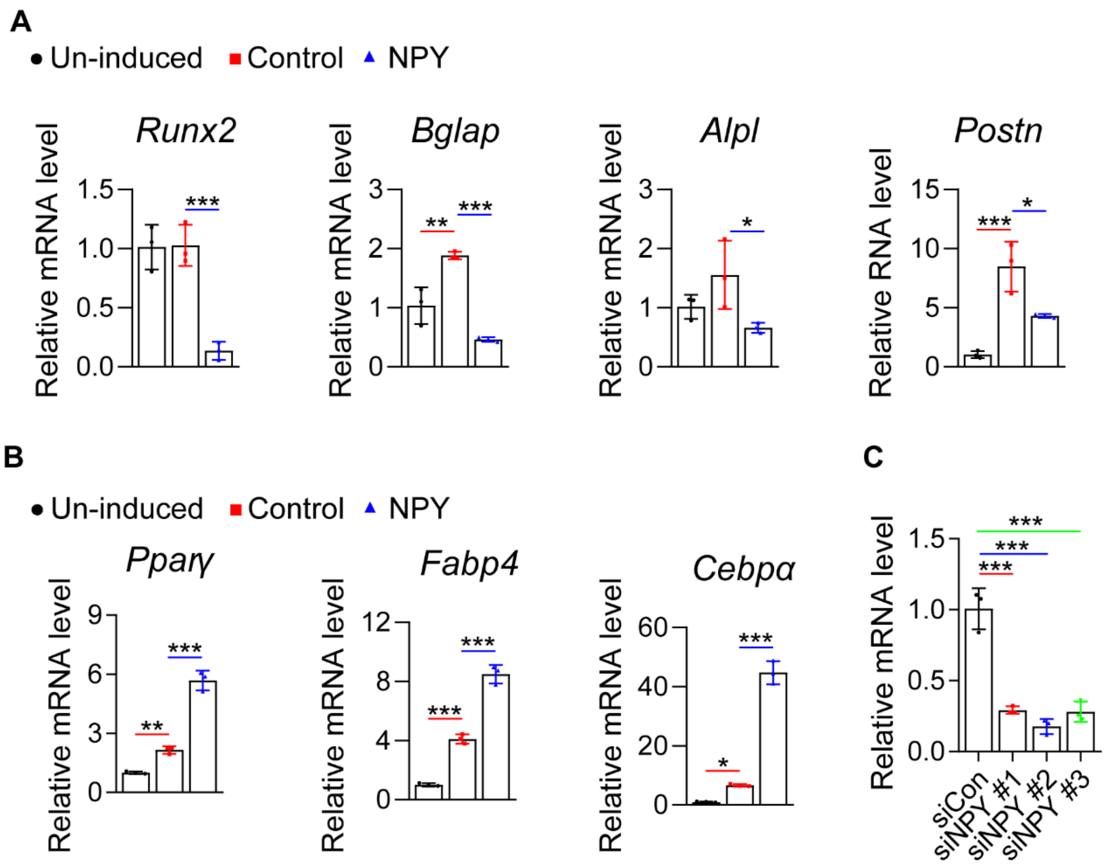

Figure S5. The expression of osteogenesis- or adipogenesis-related genes and *Npy* gene. qRT-PCR analysis of the gene expression of **A**) osteogenesis-related genes (*Runx2*, *Bglap*, *Alpl*, and *Postn*) in BMSCs under osteogenic differentiation and **B**) adipogenesis-related genes (*Pparγ*, *Fabp4*, and *Cebpα*) in BMSCs under adipogenic induction. $n = 3$ per group. **C**) qRT-PCR analysis of *Npy* expression in BMSCs transfected with the NPY-targeting siRNAs (siNPY) or the negative control siRNAs (siCon) for 24 h. $n = 3$ per group. Data are presented as mean ± SD. For panel (A)-(B): unpaired, two tailed student's *t*-test. For panel (C): one-way ANOVA combined with Bonferroni *post hoc* test. *$P < 0.05$, **$P < 0.01$, ***$P < 0.001$.

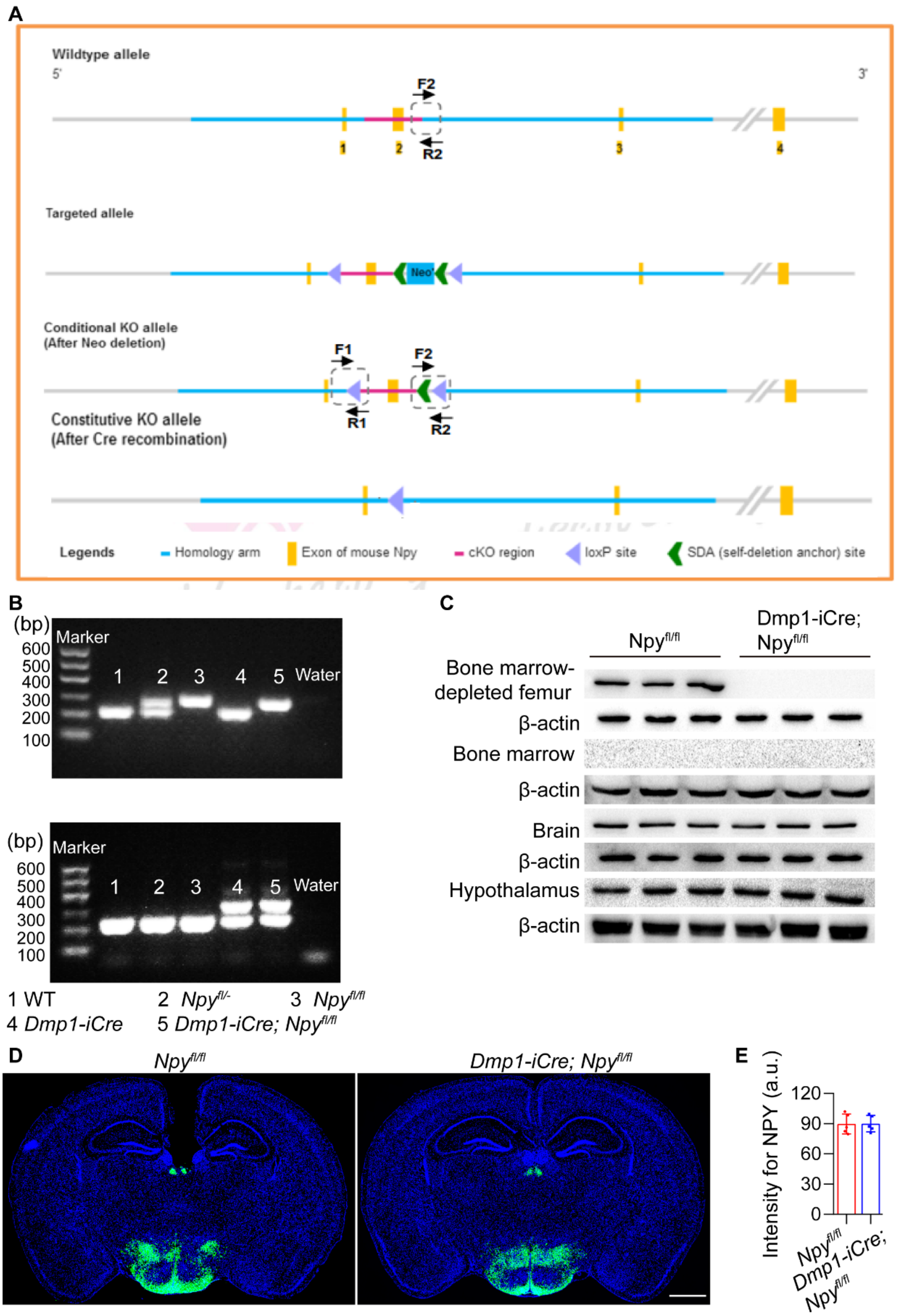

Figure S6. Conditional targeting strategy for NPY and its expression in different tissues. A) Schematic diagram of the targeting strategy for generating mice harboring the conditional *Npy* allele in which exon 2 was flanked with two *loxP* sites. **B**) PCR genotyping of wild-type mice, *Npy* $^{fl/-}$ mice, *Npy*$^{fl/fl}$ mice, *Dmp1-iCre* mice, and

Dmp1-iCre; $Npy^{fl/fl}$ mice using primers for determining the insertion of loxP (up) and *iCre* (bottom). **C**) Western blotting for NPY expression in the bone marrow-depleted femurs, whole bone marrow cells, whole brain lysate, and hypothalamus from 4-month-old male $Npy^{fl/fl}$ mice and *Dmp1-iCre*; $Npy^{fl/fl}$ mice. **D**) Immunofluorescence staining for NPY in the brain tissues and **E**) quantification of mean intensity for the NPY-positive signals. Scale bar: 500 µm. $n = 3$ per group. Data are presented as mean ± SD. Unpaired, two tailed student's *t*-test.

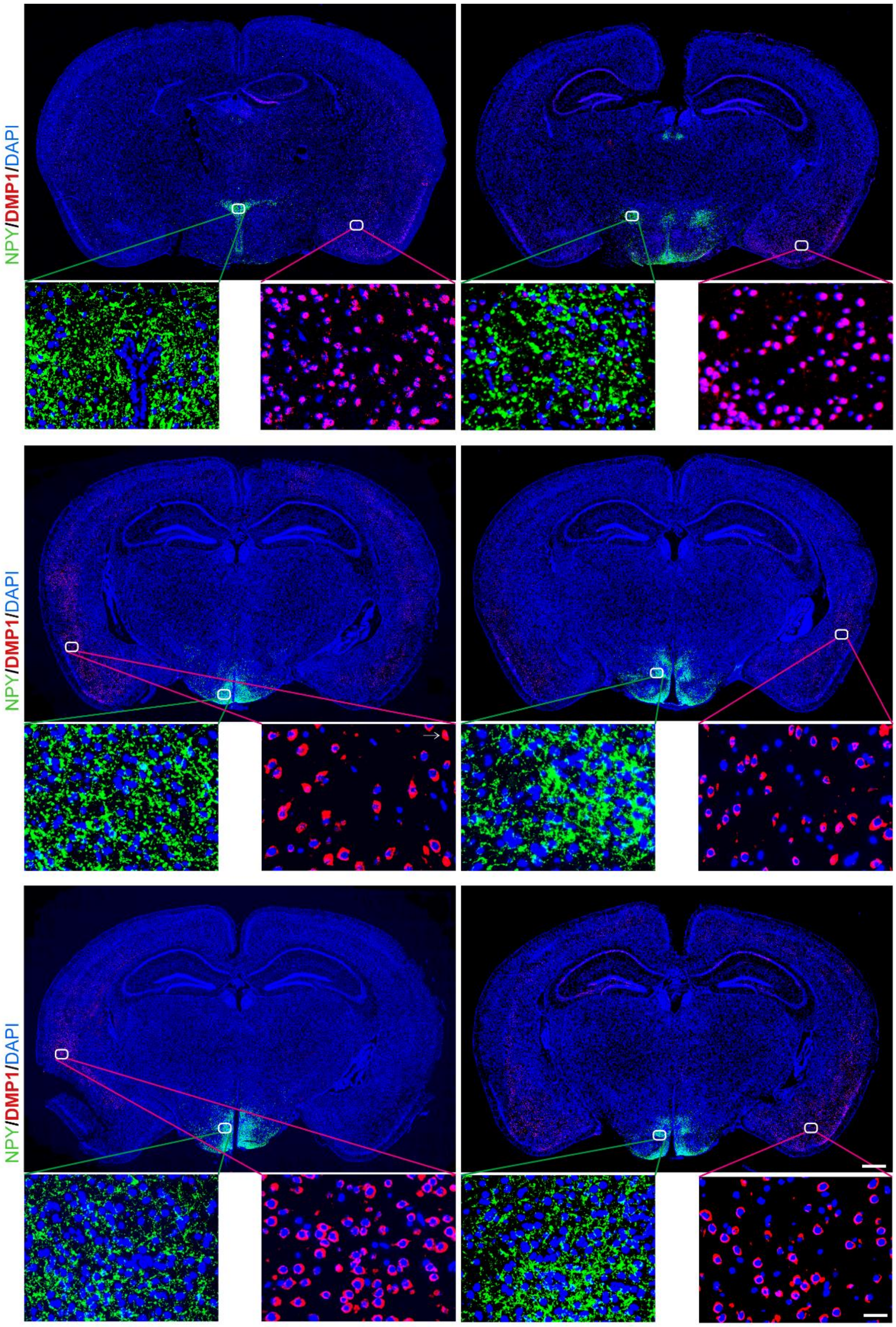

Figure S7. Detection of NPY and DMP1 expression in the brain tissues. Immunofluorescence double staining for DMP1 and NPY in the brain tissues from six 4-month-old male wild-type mice. Scale bar: 500 μm (up) or 20 μm (bottom).

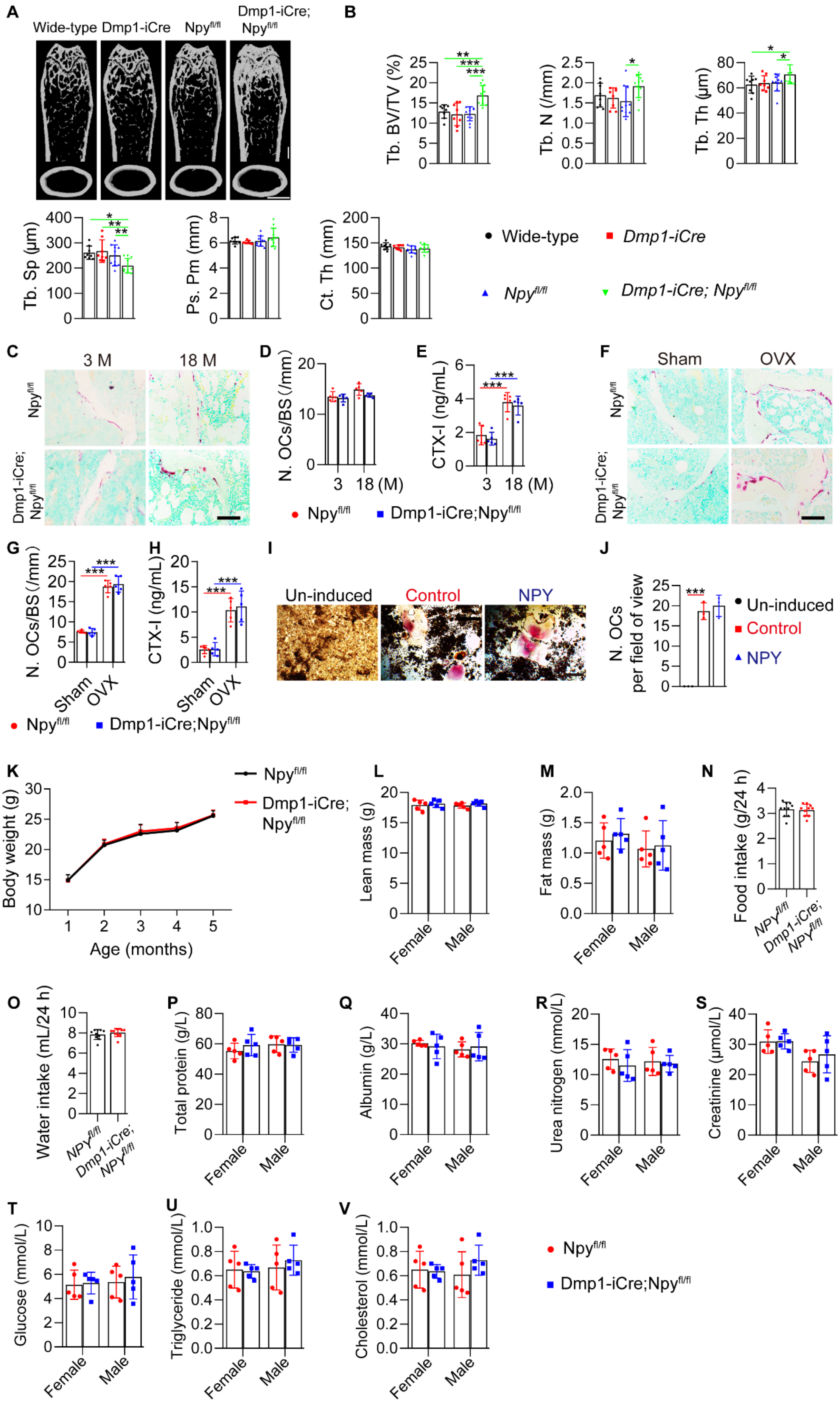
A
Wide-type
Dmp1-iCre
Npy fl/fl
Dmp1-iCre; Npy fl/fl
B
Tb. BV/TV (%)
Tb. N (/mm)
Tb. Th (μm)
Tb. Sp (μm)
Ps. Pm (mm)
Ct. Th (mm)
Wide-type
Dmp1-iCre
Npy fl/fl
Dmp1-iCre; Npy fl/fl
C
3 M
18 M
Npy fl/fl
Dmp1-iCre; Npy fl/fl
D
N. OCs/BS (/mm)
3 18 (M)
E
CTX-I (ng/mL)
3 18 (M)
Npy fl/fl
Dmp1-iCre;Npy fl/fl
F
Sham
OVX
Npy fl/fl
Dmp1-iCre; Npy fl/fl
G
N. OCs/BS (/mm)
Sham
OVX
H
CTX-I (ng/mL)
Sham
OVX
Npy fl/fl
Dmp1-iCre;Npy fl/fl
I
Un-induced
Control
NPY
J
N. OCs per field of view
Un-induced
Control
NPY
K
Body weight (g)
Age (months)
Npy fl/fl
Dmp1-iCre; Npy fl/fl
L
Lean mass (g)
Female
Male
M
Fat mass (g)
Female
Male
N
Food intake (g/24 h)
NPY fl/fl
Dmp1-iCre; NPY fl/fl
O
Water intake (mL/24 h)
NPY fl/fl
Dmp1-iCre; NPY fl/fl
P
Total protein (g/L)
Female
Male
Q
Albumin (g/L)
Female
Male
R
Urea nitrogen (mmol/L)
Female
Male
S
Creatinine (μmol/L)
Female
Male
T
Glucose (mmol/L)
Female
Male
U
Triglyceride (mmol/L)
Female
Male
V
Cholesterol (mmol/L)
Female
Male
Npy fl/fl
Dmp1-iCre;Npy fl/fl

Figure S8. Effects of osteocyte NPY deletion on bone mass, osteoclast formation and activity, body weight, daily intake of food and water, fat mass, lean mass, and metabolic indicators in mice. **A**) μCT reconstruction images and **B**) quantification of bone microarchitecture parameters in femurs from 3-month-old male *Dmp1-iCre* mice, *Npy*$^{fl/fl}$ mice, *Dmp1-iCre*; *Npy*$^{fl/fl}$ mice, and their wild-type littermates. *n* = 8-10 per group. **C**) TRAP staining images and **D**) quantification of the number of osteoclasts (N. OCs) in distal femurs from male *Npy*$^{fl/fl}$ mice and *Dmp1-iCre*; *Npy*$^{fl/fl}$ mice at different ages. Scale bar: 50 μm. *n* = 5 per group. **E**) ELISA test for serum CTX-I. *n* = 5 per group. **F**) TRAP staining images and **G**) quantification of the number of osteoclasts in distal femurs from female *Npy*$^{fl/fl}$ mice and *Dmp1-iCre*; *Npy*$^{fl/fl}$ mice subjected to OVX or sham operation. Scale bar: 50 μm. *n* = 5 per group. **H**) ELISA test for serum CTX-I. *n* = 5 per group. **I**) TRAP staining showing the effect of NPY protein on osteoclast formation of RAW264.7 cells. **J**) Quantification of the number of TRAP^{+} osteoclasts (> 3 nuclei). Scale bar: 50 μm. *n* = 3 per group. **K**) Body weight, **L**) lean mass, **M**) fat mass, and daily intake of food **N**) and water **O**) in age- and sex-matched *Npy*$^{fl/fl}$ mice and *Dmp1-iCre*; *Npy*$^{fl/fl}$ mice. *n* = 10 or 5 per group. The levels of **P**) serum total protein, **Q**) albumin, **R**) urea nitrogen, **S**) creatinine, **T**) blood glucose, **U**) total triglyceride, and **V**) total cholesterol in 3-month-old sex-matched *Npy*$^{fl/fl}$ mice and *Dmp1-iCre*; *Npy*$^{fl/fl}$ mice. *n* = 5 per group. Data are presented as mean ± SD. For panel (B): one-way ANOVA with Bonferroni *post hoc* test. For panels (J), (N), and (O): unpaired, two tailed student's *t*-test. For other dot plots: two-way ANOVA with Bonferroni *post hoc* test. ***P** < 0.05, ****P** < 0.01, *****P** < 0.001.

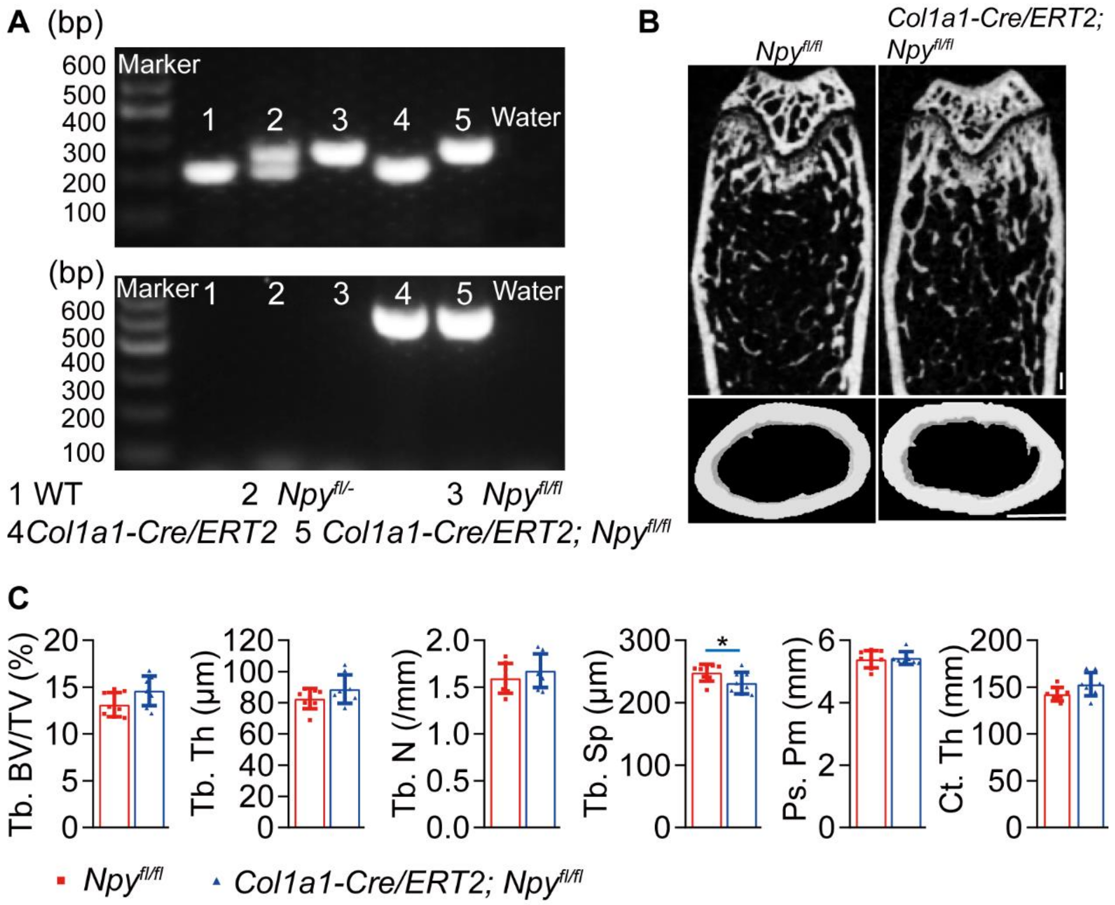

Figure S9. Effects of NPY deletion in osteoblasts on bone mass. A) PCR genotyping of wild-type mice, *Npy* $^{fl/-}$ mice, *Npy* $^{fl/fl}$ mice, *Col1a1-Cre/ERT2* mice, and *Col1a1-Cre/ERT2*; *Npy* $^{fl/fl}$ mice using primers for determining the insertion of loxP (up) and *Cre* (bottom). **B**) μCT reconstruction images and **C**) quantification of bone microarchitecture parameters in femurs from 3-month-old male *Npy* $^{fl/fl}$ mice and *Col1a1-Cre/ERT2*; *Npy* $^{fl/fl}$ mice treated with tamoxifen for consecutive 5 days and then left for two months. Scale bar: 1 mm. n = 8 per group. Data are presented as mean ± SD. Unpaired, two tailed student's t-test. *****$P < 0.05$.

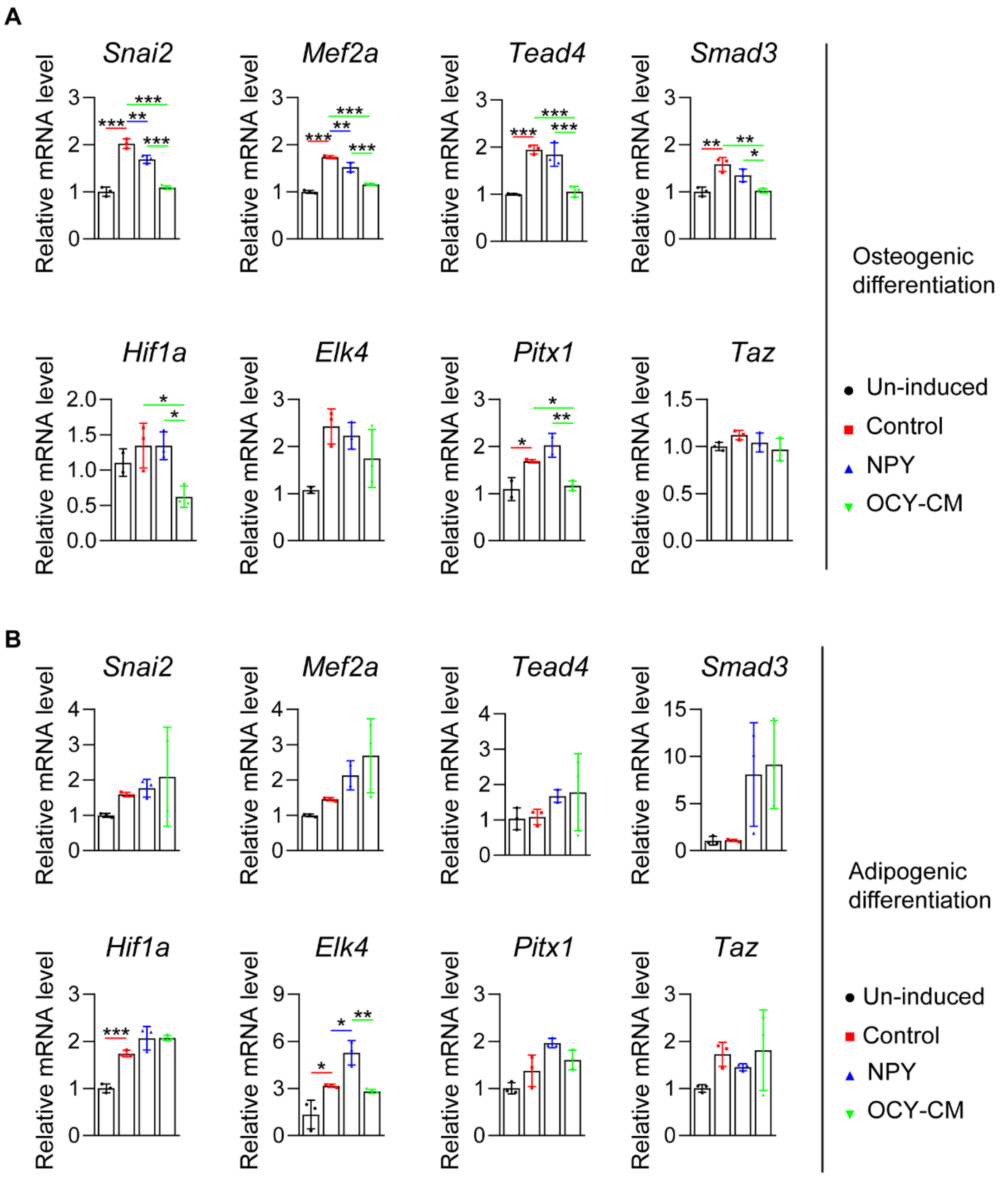

Figure S10. Effects of NPY and OCY-CM on expression of a subset of pro-osteogenic/anti-adipogenic transcription factors. qRT-PCR analysis of mRNA levels of a subset of pro-osteogenic/anti-adipogenic transcription factors in BMSCs receiving different treatments under **A**) osteogenic or **B**) adipogenic induction for 3 days. *n* = 3 per group. Data are presented as mean ± SD. Unpaired, two tailed student's *t*-test (differences between un-induced and control groups) or one-way ANOVA combined with Bonferroni *post hoc* test (differences among control, NPY, and OCY-CM groups). **P* < 0.05, ***P* < 0.01, ****P* < 0.001.

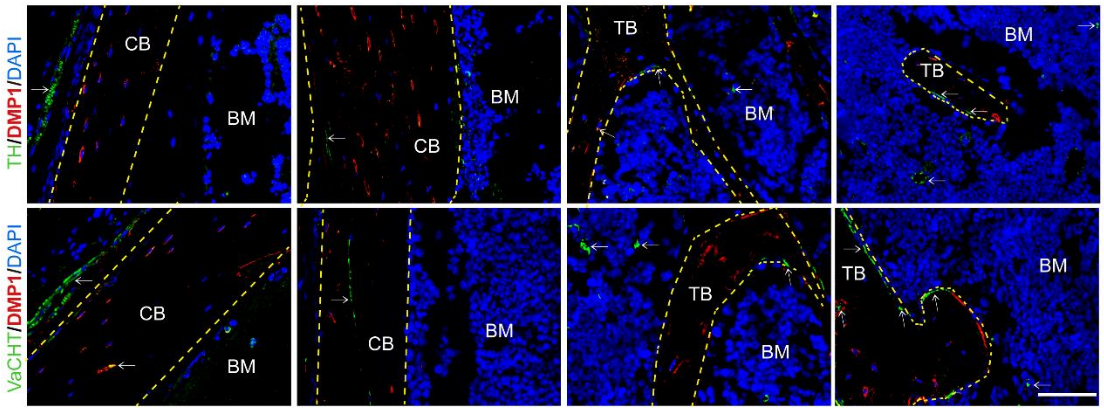

Figure S11. Detection of the sympathetic and parasympathetic fibers in the bone tissues. Immunofluorescence double staining images for DMP1/TH and DMP1/VaChT in femurs from 4-month-old male wild-type mice. White arrows indicate the presence of the TH- or VaChT-positive neuronal fibers in the periosteum, cortical bone, trabecular bone, and bone marrow. CB: cortical bone. TB: trabecular bone; BM: bone marrow. Scale bar: 50 μm.

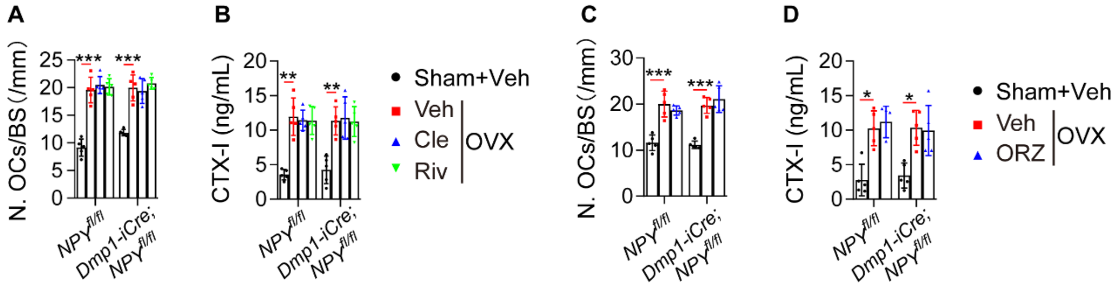

Figure S12. SNS and PSNS activation or γ-Oryzanol administration induces no obvious effects on osteoclast formation and activity. A) Quantification of the number of TRAP$^+$ osteoclasts in distal femurs from 3-month-old female *Npy*$^{fl/fl}$ mice and *Dmp1-iCre*; *Npy*$^{fl/fl}$ mice subjected to sham or OVX operation with solvent, Cle, or Riv treatments for two months. n = 5 per group. **B**) ELISA test for serum CTX-I. n = 5 per group. **C**) Quantification of the number of osteoclasts in distal femurs from 3-month-old female *Npy*$^{fl/fl}$ mice and *Dmp1-iCre*; *Npy*$^{fl/fl}$ mice subjected to sham or OVX operation with solvent or γ-Oryzanol treatments for two months. n = 5 per group. **D**) ELISA test for serum CTX-I. n = 5 per group. Data are presented as mean ± SD. Two-way ANOVA combined with Bonferroni *post hoc* test. *$P < 0.05$, **$P < 0.01$, ***$P < 0.001$.

Table S1. Primer sequences for semiquantitative PCR and qRT-PCR.

Gene	Forward (5’-3’)	Reverse (5’-3’)
Npy	CGCCACGATGCTAGGTAACAA	TGTCGCAGAGCGGAGTAGTA
Npy1R	AAATGTGTCACTTGCGGCGTTC	AGTGTTGATTCGCTTGGTCTCACTG
Npy2R	ACCGCCATCGTTGCATTGT	TCAGGGAGTATTCCCGGAAGA
Npy4R	CCAACCACTCACAGTCACCTA	GAAACAAGAGACGAACCAGATGA
Npy5R	TTTGTCACGGAGAACAATACTGC	TGCGCTTTTTCATAACAGCCAT
Npy6R	GCAAGAGCAACAACTCGGC	TGTCATGTGCATCCCTTTTACG
Sp7	ATGGCGTCCTCTCTGCTTG	TGAAAGGTCAGCGTATGGCTT
Runx2	GACTGTGGTTACCGTCATGGC	ACTTGGTTTTTCATAACAGCGGA
Bglap	CTGACCTCACAGATCCCAAGC	TGGTCTGATAGCTCGTCACAAG
Alpl	CCAACTCTTTTGTGCCAGAGA	GGCTACATTGGTGTTGAGCTTTT
Postn	TGGTATCAAGGTGCTATCTGCG	AATGCCCAGCGTGCCATAA
Pparγ	TCGCTGATGCACTGCCTATG	GAGAGGTCCACAGAGCTGATT
Fabp4	AAGGTGAAGAGCATCATAACCCT	TCACGCCTTTCATAACACATTCC
Cebpα	CAAGAACAGCAACGAGTACCG	GTCACTGGTCAACTCCAGCAC
Snai2	CAGCGAACTGGACACACACA	ATAGGGCTGTATGCTCCCGAG
Mef2a	GGAGCTGGAAATAGTCCTGTGG	GGGAGACTTTGTAGGCATGACTT
Tead1	GAGCGACTCGGCAGATAAGC	CCACACGGCGGATAGATAGC
Tead4	TCCGCCAAATCTATGACAAGTTC	CGATGTTGGTATTGAGGTCTGC
Smad3	CACGCAGAACGTGAACACC	GGCAGTAGATAACGTGAGGGA
Hif1α	ACCTTCATCGGAAACTCCAAAG	ACTGTTAGGCTCAGGTGAACT
Elk4	ATCTAACAATGGGGAGTTCAAGC	GGCTCGGCTGAGTTTATCATAAT
Pitx1	ATCGTCCGACGCTGATCTG	GCTTGTGAAGTGAGTGCGTT
Junb	TCACGACGACTCTTACGCAG	CCTTGAGACCCCGATAGGGA
Taz	ACCTGAAGTTGATGCGTTGGA	ATCCCTTTCTGGTAGACACCAT
Adrb1	TGGCTTACTGGCTTGTCTTG	TTTCCACTCGGGTCCTTG
Adrb2	GGACAACCTCATCCCTAA	AGAGTAGCCGTTCCCATA
Adrb3	CAGTCCCTGCCTATGTTTG	TTCCTGGATTCCTGCTCT
Adra1	CAAGGCCTCAAGTCCGGCCT	CTCTCGAGAAAACTTGAGCAG
Adra2	GTGACACTGACGCTGGTTTG	CCAGTAACCCATAACCTCGTTG
Chrna1	TCTCAAGCAAAAAGTGGTC	ATTCCGAGATCTGCCTGTCT
Chrna2	CTCCCATCCTGCTTTCCAG	GTTTGAACAGGCGGTCCTC

Chrna3	CGCCTGTTCCAGTACCTGTT	CAGAGGGTTTCCATTTCAGC
Chrna4	CTGTTCTATGATGGGCGTGTG	GATGAAGGCGTAGGTGATGTC
Chrna5	CCAGCTAATGACCACCAACG	GCTGCGTCCAAGTGACAGT
Chrna6	CCTGCACTCCGGTTTATGTC	CAGCCACAGATTGGTCTCCA
Chrna7	ACAATACTTCGCCAGCACCA	AAACCATGCACACCAATTCA
Chrna9	CAATGCTCTGCGTCCAGTAG	ACACCAGATCGCTGGGAATC
Chrna10	TCTGCTCCTGCTCTTTCTCC	CCACAGGTACAAGGTCAGCA
Chrnβ1	TGATGTGGTGCTGCTGAACAA	CAACGTCGAAATTTCCGTCAT
Chrnβ2	CGAAGTGAAGATGATGACCAGA	GTCCCAAAGACACAGACAAAGA
Chrnβ3	CGATGGAACGGAGAGTAAGG	AGAGGAAGATGCGGTCAAGA
Chrnβ4	GCAAGCCACTCTTCTACACCAT	GCACATTGAGGACACACACAGT
Chrnγ	GATGCAATGGTGCGACTATCGC	GCCTCCGGGTCAATGAAGATCC
Chrnδ	GAGAACGGTGAGTGGGAGATAGT	CTTGGAGATAAGCAGCAGGAAG
Chrnξ	AATGAAGAGCTTAGCCTGTA	TACACCTGCAAAATCGTCCT
Chrm1	GCAGCAGCTCAGAGAGGTCACAG	GATGAAGGCCAGCAGGATGG
Chrm2	GCGGATCCTGTGGCCAACCAAGAC	CGAATTCACGATTTTGCGGGCTA
Chrm3	AAGGCACGAAACGGTCATCT	GCAAACCTCTTAGCCAGCGT
Chrm4	AGCCGCAGCCGTGTTCACAA	TGGGTTGAGGGTTCGTGGCT
Chrm5	GTCTCCGTCATGACCATACTCTA	CCCGTTGTTGAGGTGCTTCTAC
Gapdh	AGGTCGGTGTGAACGGATTTG	TGTAGACCATGTAGTTGAGGTCA
β-actin	GGCTGTATTCCCCTCCATCG	CCAGTTGGTAACAATGCCATGT
